# Mass spectrometry imaging reveals differential localization of natural sunscreens in the mantle of the giant clam *Tridacna crocea*

**DOI:** 10.1038/s41598-019-57296-9

**Published:** 2020-01-20

**Authors:** Naoko Goto-Inoue, Tomohiko Sato, Mizuki Morisasa, Hiroshi Yamashita, Tadashi Maruyama, Hiroki Ikeda, Ryuichi Sakai

**Affiliations:** 1 Department of Marine Science and Resources, College of Bioresource Sciences, Nihon University, 1866 Kameino, Fujisawa, Kanagawa, 252-0880 Japan; 2 Research Center for Subtropical Fisheries, Seikai National Fisheries Research Institute, Japan Fisheries Research and Education Agency, Ishigaki, Okinawa 907-0451 Japan; 3 School of Marine Biosciences, Kitasato University, 1-15-1, Kitazato, Minami, Sagamihara, Kanagawa 252-0374 Japan; 4 Faculty and Graduate School of Fisheries Sciences, Hokkaido University, 3-1-1 Minato-cho, Hakodate, 041-8611 Japan

**Keywords:** Biochemistry, Biological techniques, Chemical biology

## Abstract

Giant clams have evolved to maximize sunlight utilization by their photosymbiotic partners, while affording them protection from harmful ultraviolet (UV) light. The presence of UV absorbing substances in the mantle is thought to be critical for light protection; however, the exact localization of such compounds remains unknown. Here, we applied a combination of UV liquid chromatography (LC), LC-mass spectrometry (MS), MS imaging, and UV micrography to localize UV absorbing substances in the giant clam *Tridacna crocea*. LC-MS analysis revealed that the animal contained three classes of mycosporines: progenitor, primary, and secondary mycosporines. MS imaging revealed that primary and secondary mycosporines were localized in the outermost layer of the mantle; whereas progenitor mycosporines were distributed throughout the mantle tissue. These findings were consistent with the results of UV micrography, which revealed that the surface layer of the mantle absorbed UV light at 320 ± 10 nm. This is the first report indicating that progenitor and primary mycosporines are metabolized to secondary mycosporines by the giant clam and that they are differentially localized in the surface layer of the mantle to protect the animal from UV light.

## Introduction

Tridacnid giant clams are the largest bivalve group found in coral reef areas. Among them, *Tridacna crocea* (*T*. *crocea*), commonly referred to as “boring clam”, is found preferentially in shallow reef areas^1^. Although potentially harmful ultraviolet (UV)-B radiation (300–320 nm) is high in shallow water, it remains unclear how sessile organisms including giant clams protect themselves from strong sunlight and prevent it from having an excessive impact on their physiology and ecology. Many invertebrates harbouring symbiotic algae contain mycosporine-like amino acids (imino-mycosporines) and oxo-mycosporines (we collectively denote these synonyms as ‘mycosporines’)^2^, which absorb UV-B light very efficiently and convert it into heat^3,4^. Giant clams including *T*. *crocea* harbour symbiotic algae known as “zooxanthellae”^5^ that belong to the dinoflagellate family of Symbiodiniaceae. This family includes highly diverse members, ranging from clades A to I^6^, with some of the clades having been re-classified into genera^7^. Interestingly, some members of clade-A zooxanthellae (currently the genus *Symbiodinium*) possess the biosynthetic machinery for mycosporine production^8^. *T*. *crocea* clams harbour a *Symbiodinium* symbiont^9–11^. Importantly, the mycosporines shinorine, mycosporine-Gly, and palythine have been detected in the mantle of *T*. *crocea* and their concentrations have been reported to be higher at or near the surface of the mantle^12^. Because the composition of mycosporines varied individually, and they were not detected in isolated zooxanthellae, these mycosporines were suspected to originate from the animal’s diet rather than the *Symbiodinium* symbiont^12^. However, this result remains to be confirmed as the *Symbiodinium* that inhabits the clam has later been shown to contain mycosporines^13^ and to retain a biosynthetic gene for mycosporine production^8^. Thus, the true origin of mycosporines in the clam is still elusive. Here, we tried to determine the composition and sub-tissue localization of mycosporines in the giant clam by applying liquid chromatography (LC)-UV, LC-tandem mass spectrometry (MS/MS), MS imaging, and UV micrography. The sub-tissue localization of structurally different mycosporines sheds light on their biosynthesis, transportation, and spatial expression in the clam.

## Results

### Identification and semi-quantitative comparison of mycosporines from *T*. *crocea* extracts

**Figure 1:**
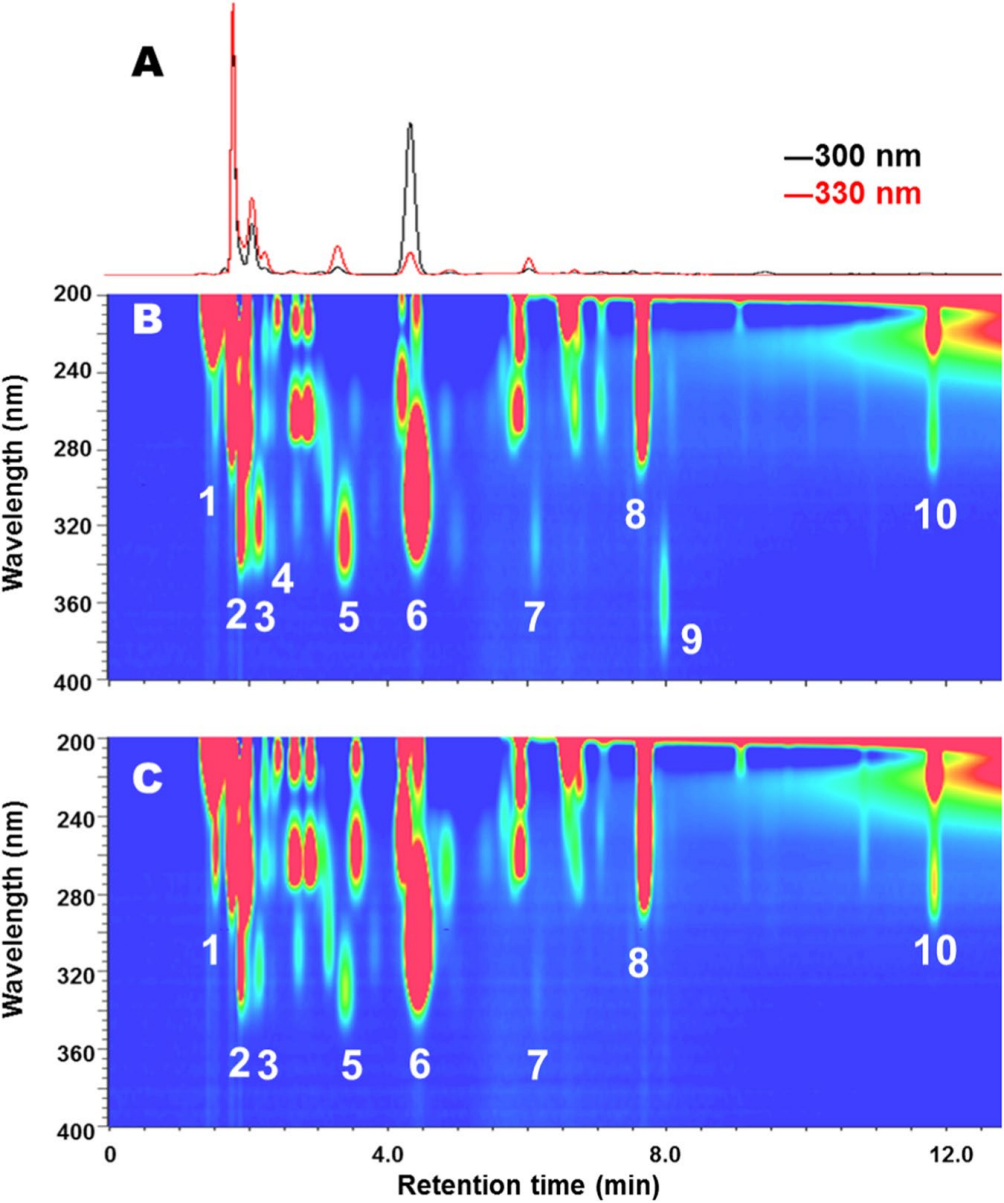
PDA chromatograms of *T*. *crocea* extracts. PDA chromatograms of clam extracts describing (**A**) traces for 300 and 330 nm absorptions, (**B**) two dimensional chromatogram for the epidermal layer (EL) and (**C**) that for the inner layer (IL) between 200 and 400 nm. Peak annotation: (1) trigonelline, (2) palythine, (3) unidentified mycosporine A, (4) asterina-330, (5) porphyra-334 and palythinol (overlapped) (6) mycosporine-Gly, (7) unidentified mycosporine B, (8) inosine, (9) usujirene/palythene, (10) tryptophan.

**Figure 2:**
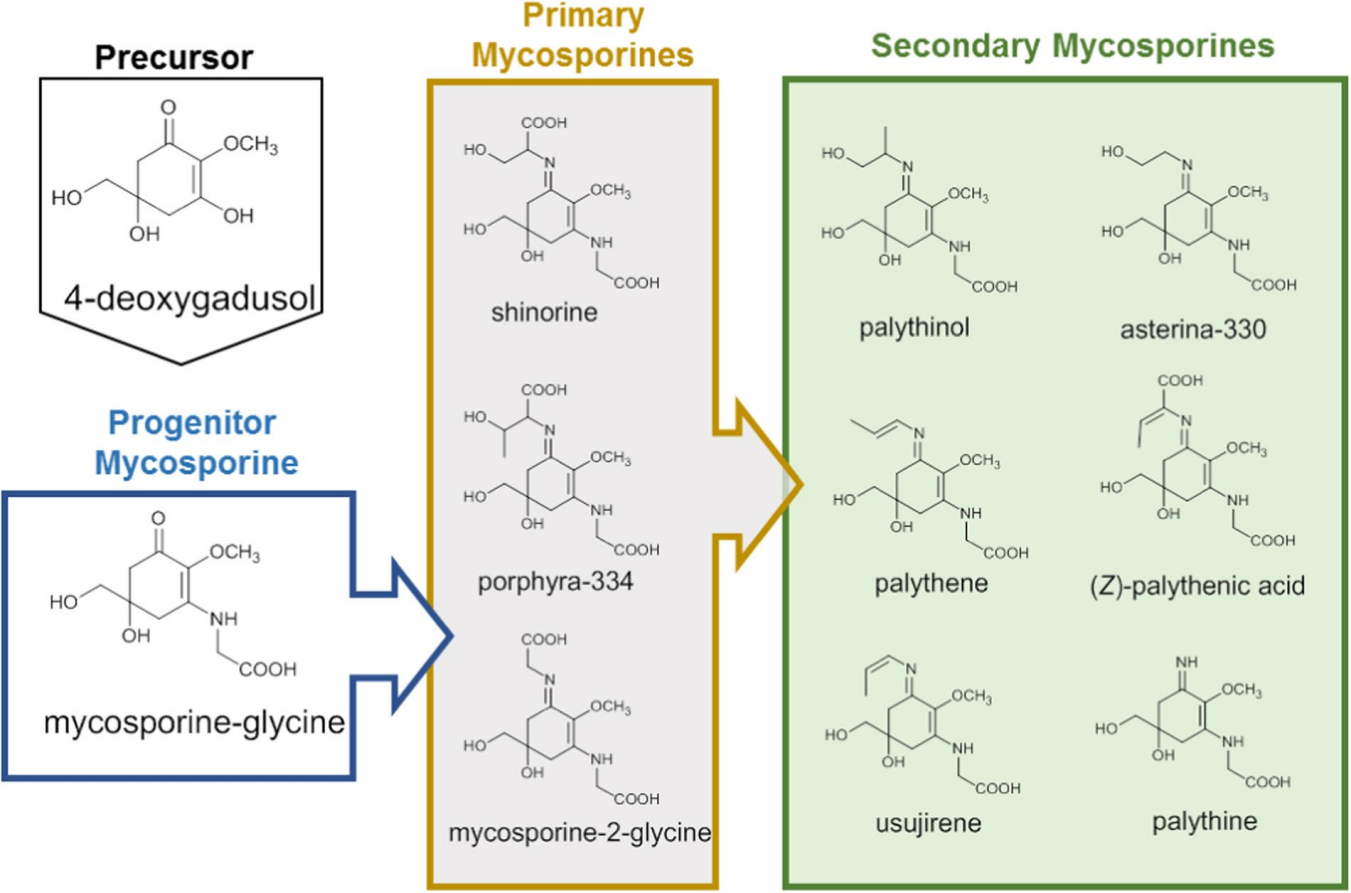
Detected mycosporines. Structures and biosynthetic relationships of mycosporines identified in the present work.

We first determined the chemical composition of mycosporines and related compounds in the mantle of *T*. *crocea*. Giant clams have a tubule that contains zooxanthellae, and a branch of it reaches into the upper levels of the inner fold in the siphonal mantle^14^. Thus, we dissected the frozen mantle specimens into an exposed epidermal layer, which contained zooxanthellae at high density, and a deeper inner layer adjacent to the epidermal layer with a much lower density of zooxanthellae. We denoted these layers as epidermal layer (EL) and inner layer (IL), respectively. The two parts were extracted separately and subjected first to LC-UV analysis using a photodiode array (PDA) detector, and then analysed by LC-MS/MS (Fig. 1). The detected mycosporines are listed in Table 1. As shown by the PDA chromatographs in Fig. 1, the aqueous extracts of the two parts contained various mycosporines and related compounds. LC-MS/MS analysis of these extracts led to the identification of 4-deoxygadusol and mycosporine-Gly, besides eight mycosporine-amino acids (MAAs), including shinorine, porphyra-334, mycosporine-2-Gly, palythine, asterina-330, palythinol, palythenic acid, and usujirene/palythene (stereoisomers). 4-Deoxygadusol is a common precursor of mycosporines and is formed both through the shikimic acid and pentose phosphate pathways^15,16^; whereas mycosporine-Gly serves as progenitor for all imino-mycosporines (MAAs)^16^ (Fig. 2).

**Table 1:** Mycosporines detected in the extracts of *T*. *crocea*^*a*^.

Category	Compound	Formula	Theoretical [M + H]^+^, m/z	Ion observed [M + H]^+^, *m*/*z*	
				LC-MS	FT-ICR MS imaging,
Precursor	4-deoxygadusol	C_8_H_12_O_5_	189.0753	189.0763	n.d.
Progenitor	mycosporine-Gly	C_10_H_15_NO_6_	246.0972	246.0977	246.0995
Primary	shinorine	C_13_H_20_N_2_O_8_	333.1292	333.1297	333.1282
	porphyra-334	C_14_H_22_N_2_O_8_	347.1449	347.1454	347.1482
	mycosporine-2-Gly	C_12_H_18_N_2_O_7_	303.1187	303.1192	303.1153
Secondary	asterina-330	C_12_H_20_N_2_O_6_	289.1394	289.1399	289.1425
	palythinol	C_13_H_22_N_2_O_6_	303.1551	303.1556	303.1582
	palythine	C_10_H_16_N_2_O_5_	245.1132	245.1137	245.1153
	usujirene/palythene	C_13_H_20_N_2_O_5_	285.1445	285.1450	285.1473
	palythenic acid	C_14_H_20_N_2_O_7_	329.1343	329.1348	329.1376

^*a*^Spectral and chromatographic data are provided in the Supplementary information available online.

Here, we denoted mycosporine-Gly as a ‘progenitor mycosporine’^17^ and categorized 4-deoxygadusol as a precursor of mycosporines. Shinorine, prophyra-334, and mycosporine-2-Gly are called primary mycosporines because they can be biosynthesized primarily by *Symbiodinium* algae^17^. Instead, palythine, asterina-330, palythinol, palythenic acid, and usujirene/palythene are called secondary mycosporines because they are processed from primary mycosporines^17^. Other UV absorbers including trigonelline, inosine, tryptophan, and several unidentified substances were also found. In Fig. 1, peaks **3** and **7** are denoted as unidentified mycosporines A and B, respectively, since no standard mycosporine in our library corresponded to them. Isolation and structure determination of these compounds are in progress and will be reported in due course.

**Figure 3:**
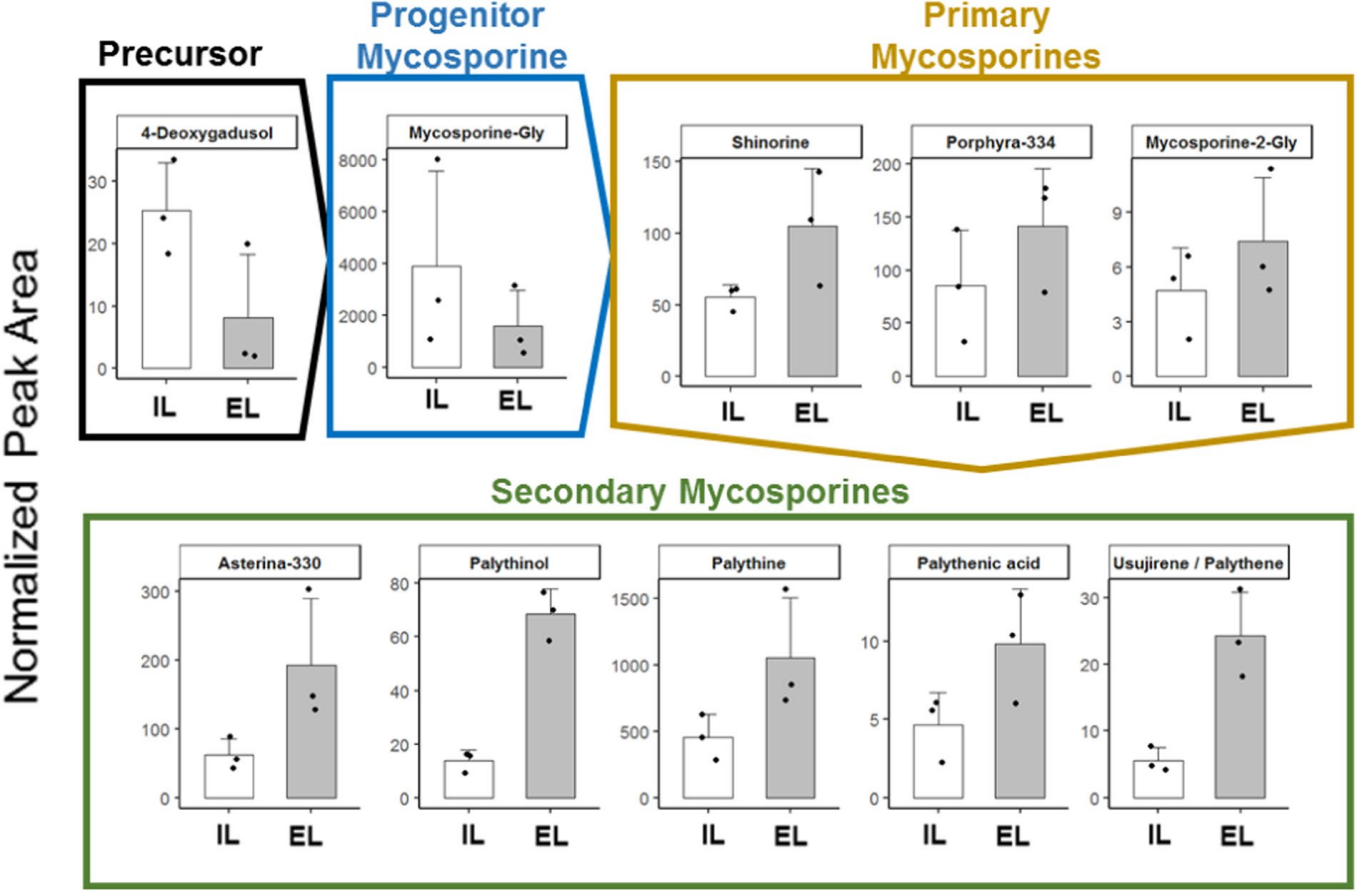
Semi-quantitative comparison of mycosporines between EL and IL. Semi-quantitative LC-MS analysis of mycosporines. Averaged relative peak area (histograms), data points (dots), and standard deviation (bar, n = 3) are plotted.

LC-MS/MS followed by principal component analysis (PCA, see Supplementary Figs. 1S and 2S) indicated a significant difference between the metabolic profiles of the EL and IL, suggesting large physiological differences, especially in the ability to minimize UV damage, between these parts. Semi-quantitative LC-MS/MS detected large quantities of 4-deoxygadusol and mycosporine-Gly in the IL. In contrast, the more complex primary and secondary mycosporines were found at higher concentrations in the EL (Fig. 3). The UV-A absorbers usujirene/palythene were present mostly in the EL but at a low concentration (Fig. 3, Supplementary Figs. 3S and 4S).

### MS imaging reveals the localization of mycosporines

**Figure 4:**
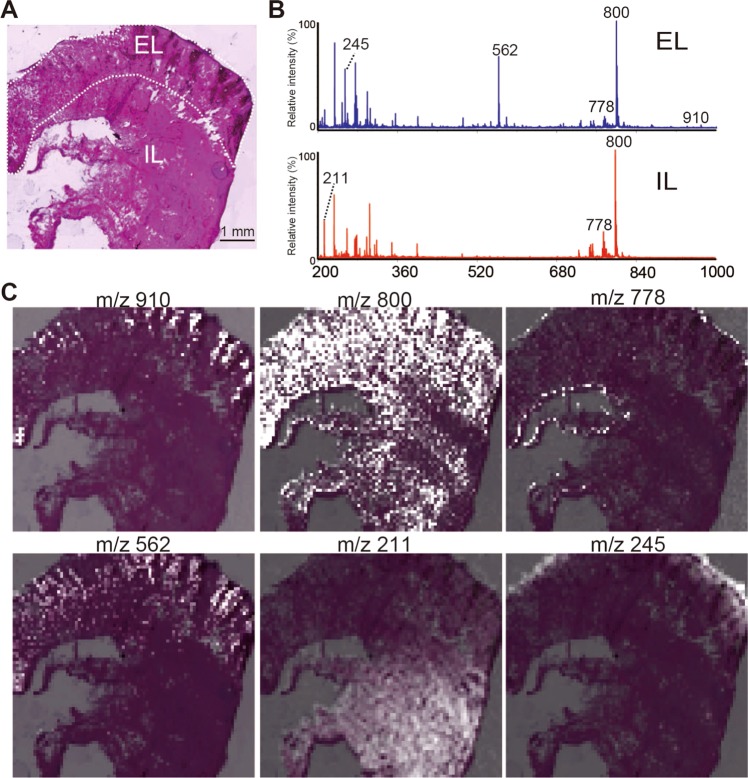
MALDI MS imaging results of *T*. *crocea*. (**A**) Haematoxylin and eosin (HE) staining of *T*. *crocea* frozen sections. (**B**) Summed mass spectral observations of EL and IL. (**C**) Mass images of each major peak at *m/z* 910, 800, 778, 562, 211, and 245 superimposed on the HE-stained image.

Haematoxylin and eosin (HE) staining of frozen tissue sections revealed flocks of zooxanthellae at the edges of the EL and the mantle area of *T*. *crocea* (IL) (Fig. 4A). Matrix-assisted laser desorption/ionization (MALDI)-time of flight (TOF) MS analysis of the tissue sections indicated that the summed-spectra of the imaged areas contained some dominant peaks such as the one at *m*/*z* 800 (Fig. 4B). Peaks specific to the EL were found at *m*/*z* 910, 562, and 245; the one specific to the IL was recorded at *m*/*z* 211. Superimposing MS images of the sections with the HE results revealed the tissues responsible for the above ions (Fig. 4C). The EL-specific ions *m*/*z* 910 and 562 were distributed in the algal cells; whereas the one at *m*/*z* 245 was detected only in the outer layer of the epithelium. A large common ion at *m*/*z* 800 was localized throughout the tissue. The peak at *m*/*z* 778 was also distributed solely in the outer epithelium. Finally, the IL-specific peak at *m*/*z* 211 was distributed in the inner tissue of the section.

**Figure 5:**
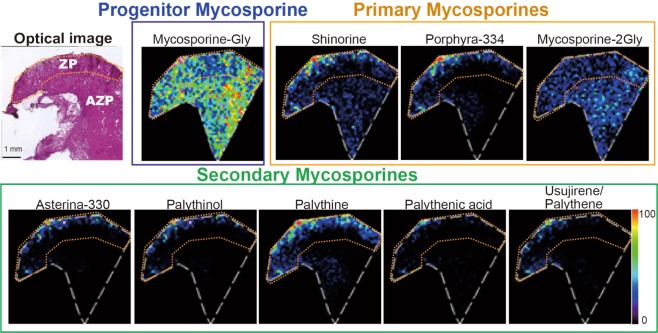
MALDI FT-ICR MS imaging of nine mycosporines. MALDI FT-ICR MS imaging of *T*. *crocea* frozen sections for selected mycosporines. Progenitor mycosporine-Gly, three primary mycosporines (shinorine, porphyra-334, mycosporine-2-Gly), and five secondary mycosporines (asterina-330, palythinol, palythine, usujirene/palythene, and palythenic acid) were visualized.

Based on the above LC-MS/MS and UV-high-performance liquid chromatography (HPLC) data (Table 1, Supplementary Table 1S), the ion at *m*/*z* 245 was assumed to be palythine. The putative identification of palythine encouraged us to explore the presence of other mycosporines in the specimen using a MALDI Fourier transform-ion cyclotron resonance (FT-ICR) MS system that allowed us to obtain the exact masses of the observed ions. Combined with the above LC-MS/MS results, this approach could enable the ready identification of mycosporine peaks on the tissue. We first obtained the spectra for pure mycosporine/MAAs using the FT-ICR MS imaging system and confirmed that all gave a clear [M + H]^+^ molecular ion (Table 1, Supplementary Fig. 5S). We then conducted MALDI FT-ICR MS imaging of mycosporine/MAAs at a sub-tissue level. Ions were selected on the basis of these and LC-MS/MS results. We failed to detect the molecular ion of 4-deoxygadusol because it was below the detention limit; however, the localization of other mycosporine/MAAs is shown in Fig. 5. Interestingly, the progenitor mycosporine-Gly localized differently from MAAs as it was present uniformly throughout the tissue sections, with slightly higher signal intensity in the IL than in the EL. The primary mycosporines shinorine and porphyra-334 were highly abundant at the EL but much less so in the IL. In comparison, mycosporine-2-Gly localized rather widely in a pattern similar to that of mycosporine-Gly. Among secondary mycosporines, palythine gave the highest intensity with clear localization in the outer layer of the epithelium. Other secondary mycosporines, such as asterina-330, palythinol, palythenic acid, and *m*/*z* 285 (for usujirene/palythene plus unidentified mycosporine) were detected only weakly, but shared the same localization pattern as palythine.

### Localization of sunscreen compounds by UV imaging

**Figure 6:**
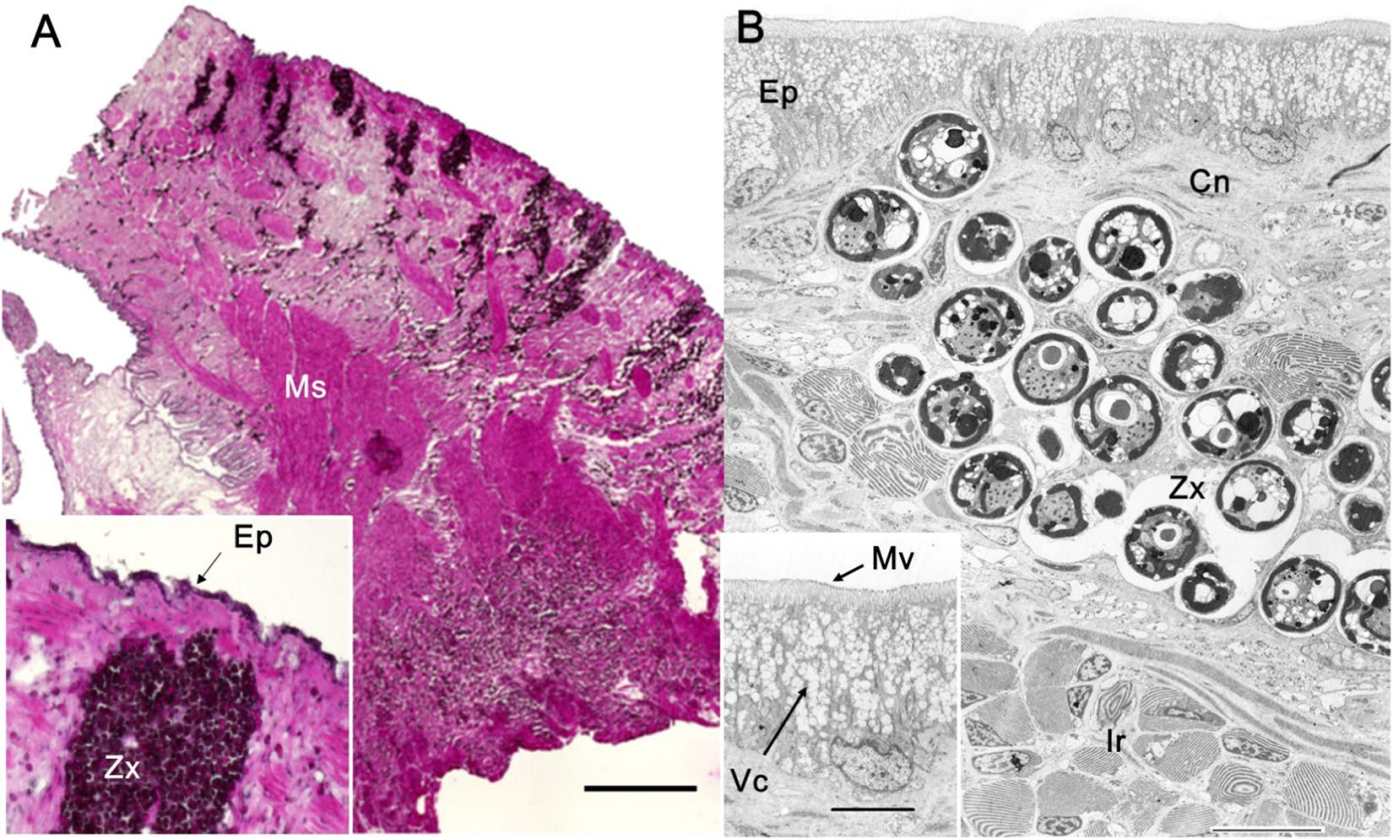
Light and electron micrographs of the mantle of *T*. *crocea*. (**A**) Light micrographs of formaldehyde-fixed and HE-stained frozen sections of the mantle. The insert shows the surface area of the mantle at a higher magnification. (**B**) Transmission electron micrograph of a glutaraldehyde- and OsO_4_-fixed mantle section. The insert shows the epithelial cell layer at a higher magnification. This data was obtained from different individuals of *T*. *crocea*. **Ep**, epithelium; **Ir**, iridophore; **Cn**, connective tissue; **Ms**, muscle; **Mv**, microvilli on the surface of the epithelial cell; **Vc**, vacuoles in the epithelial cell. **Zx**, zooxanthellae. Bar in **A**, 1 mm; in **B**, 10 μm; in the insert in **B**, 5 μm.

Prior to UV imaging, we examined the detailed tissue structure of the mantle. In both light and electron micrographs, a single epithelium cell layer was observed in the outermost layer (Fig. 6). Epithelial cells displayed microvilli on the outer surface and several vacuoles in the cytoplasm, as also reported previously^18^. Below the epithelium cell layer, micrographs revealed the presence of connective tissue (Fig. 6B), zooxanthellae (Fig. 6A,B), and unique iridophores (Fig. 6B), as reported previously^18^. Muscle cells were observed dispersed in the EL but aggregated in the IL (Fig. 6A).

**Figure 7:**
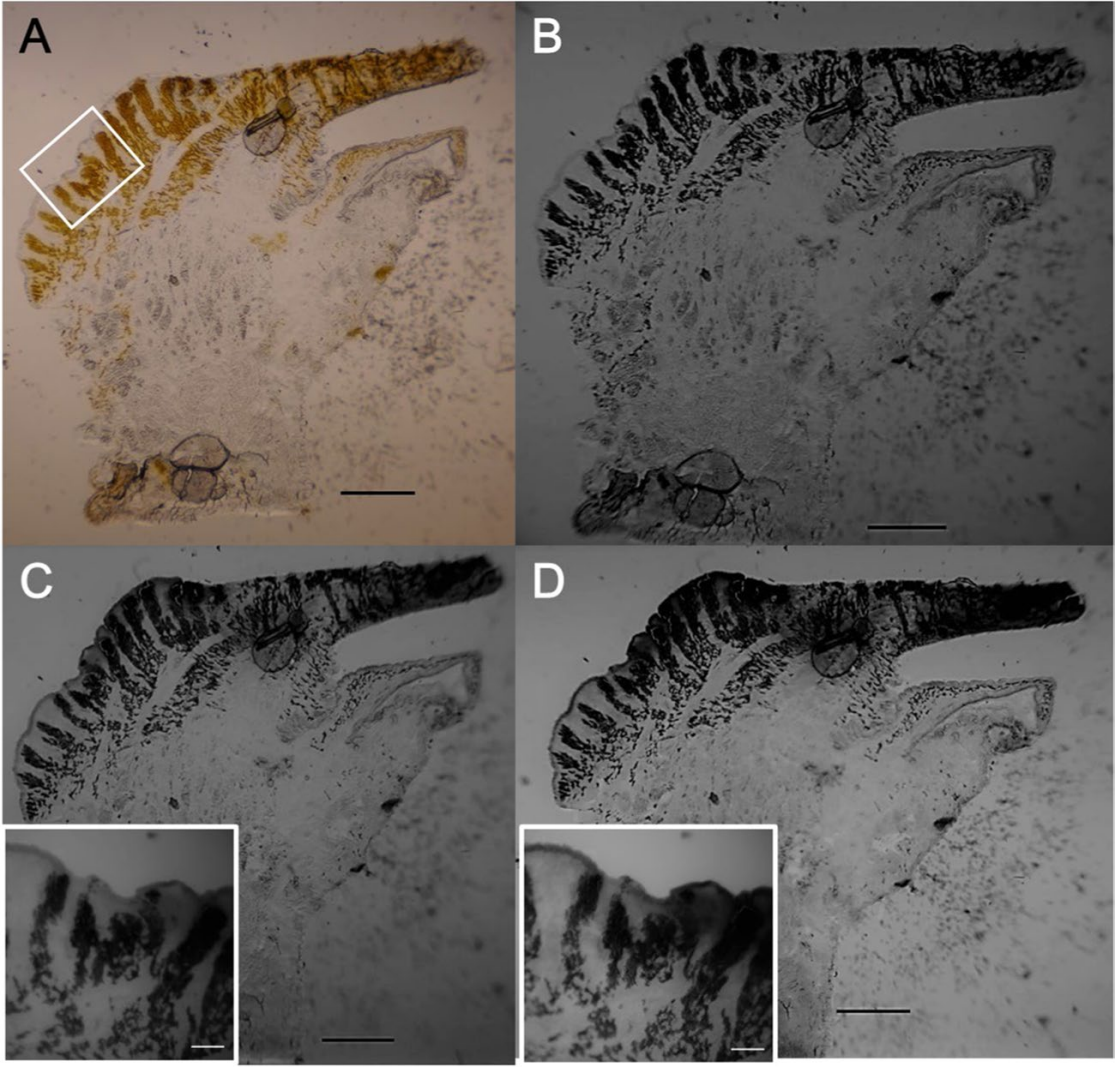
UV photographs of a frozen section of the *T*. *crocea* mantle. (**A**) Colour photograph of the section through a white fluorescent tube light. (**B**) UV photograph of the section through UV-B light source at 365 ± 10 nm. (**C**) UV photograph of the section through UV-B light source at 337 ± 10 nm. (**D**) UV photograph of the section through UB-B light source at 320 ± 10 nm. The inserts in **C** and **D** show the magnified surface area of the mantle, which corresponds to the white square in **A**, at 337 and 320 nm, respectively. These UV photographs were obtained from the same *T*. *crocea* individual as that shown in Fig. 6A. Bars in **A**, **B**, **C**, and **D**, 1 mm; bars in the inserts in **C** and **D**, 200 μm.

To determine the localization of UV absorption in the clam’s tissue, we used UV micrography. None of the examined UV light appeared to be absorbed by the IL (Fig. 7). Instead, in the EL, zooxanthella cells appeared brown under visible light and dark under UV light at all examined wavelengths, indicating that the zooxanthellae strongly absorbed UV light. Notably, except for the zooxanthellae, the animal tissue in the EL was mostly transparent at 365 ± 10 nm (Fig. 7B), while the same regions absorbed UV light moderately at 337 ± 10 nm and strongly at 320 ± 10 nm (Fig. 7C,D). The absorbance was most prominent in the outer surface at approximately 50 μm of thickness; but could be extended into the deeper layer, to 100 μm or sometimes even 500 μm below the surface (Fig. 7).

## Discussion

Mycosporines have long been studied as multi-functional marine secondary metabolites and are thought to play a prominent role as sunscreen in marine organisms^3^. The widespread distribution of mycosporines from bacteria, microalgae, and invertebrates to fish suggests their translocation via the food web or symbiosis^19^. The actual role as sunscreen and true origin of mycosporines, however, remain elusive because of a lack of compelling evidence. In the present study, we employed the giant clam *T*. *crocea* as a model to determine the presence of mycosporines and related molecules using LC-MS/MS and their localization at sub-tissue level using a combination of UV and MS imaging techniques. We chose the giant clam because of its strict dependence on symbiosis with the zooxanthella symbiont and, consequently, the impact of mycosporines on its life style. The giant clam is also an ideal model for imaging studies as its siphonal mantle is large and the section is nearly transparent.

Metabolic profiles of mycosporines differed significantly between the EL and IL, suggesting that they exert differential physiological functions. PCA analyses revealed that mycosporines could represent the main discriminants among these two parts. Two progenitor mycosporines were present at a higher concentration in the IL, while all the eight imino-mycosporines were more abundant in the EL. The observed mycosporine profiles shared a similar trend as that recorded in 48 species of Caribbean corals^20^, where mycosporine-Gly was generally present at the highest concentration in both the host and symbiont fractions. However, mycosporines in *T*. *crocea* revealed a more diverse distribution between the precursor mycosporine 4-deoxygadusol and the highly modified nine other mycosporines, as detected by LC-MS and MS imaging. This suggested the presence of diverse biosynthetic machineries in the clam-zooxanthellae consortium. It is also possible that the progenitor mycosporines in the clam are supplied either internally (synthesized by the clam) or externally (from symbionts and/or foods), and can then be converted to other imino-mycosporines in the clam’s tissue. For example, the coral *Acropora digitifera* possesses a gene cluster for shinorine biosynthesis, meaning that mycosporine synthesis in this coral species is not symbiont-dependent^21,22^. At present, mycosporine biosynthetic genes have not been reported in giant clams.

It is generally accepted that certain types of *Symbiodinium* can biosynthesize mycosporines. The presence of a biosynthetic gene set in clade-A, but not in clade-C zooxanthellae has been reported^8^. It has also been shown that cultured clade-A zooxanthellae can biosynthesize four primary mycosporines: shinorine, porphyra-334, mycosporine-Gly, and mycosporine-2-Gly^17^. Of note, shinorine and porphyra-334 constitute the most abundant mycosporines in cultured clade-A algae^13^. Shick *et al*. have reported the conversion of primary to secondary mycosporines in the coral *Stylophora pistillata* upon various light stimuli, suggesting that primary mycosporines could be converted to secondary ones by enzymes from either zooxanthellae or the host organism^17^. Furthermore, light-stimulated two-step interconversion of MAAs in the red-tide dinoflagellate *Alexandrium tamarense* was reported, in which the primary mycosporines once biosynthesized by the algae were interconverted, presumably enzymatically, to other secondary MAAs, followed by accumulation of the converted compounds in the cells^23^. Based on this and on the results herein presented, we may conclude that light-induced interconversion between primary and secondary mycosporines has evolved both in symbiotic and free living microalgae^24^.

MS imaging in the present study expanded on the above evidence by showing differential localization of mycosporines in the tissue of the giant clam. Accordingly, we demonstrate for the first time that progenitor mycosporines such as mycosporine-Gly; primary mycosporines, such as shinorine, porphyra-334, and mycosporine-2-Gly; and secondary mycosporines, including asterina-330, palythinol, palythine, palythenic acid, and usujirene/palythene exhibited discrete localization in the tissue of *T*. *crocea*. Notably, secondary mycosporines including palythine were found to localize in the epithelium. Because palythine (λ_max_ = 320 nm) was the second most abundant mycosporine in the clam, we believe its abundance and localization close to the surface may imply a major role as a sunscreen agent. This result was in good agreement with UV micrograph imaging data, whereby light at 320 nm was most efficiently absorbed on the surface of the mantle tissue. Moreover, the observed distinct distribution of mycosporine-Gly was intriguing as its structure differs from that of palythine by an imino group instead of a carbonyl group. This structural modification causes a bathochromic shift of 10 nm. It may also determine the widespread distribution of mycosporine-Gly, and its role as a precursor of other imino-mycosporines found in this study. The conversion of primary mycosporines into secondary mycosporines upon irradiation with UV light has been demonstrated experimentally in the coral *S*. *pistillata*^17^. It is therefore tempting to speculate that the primary mycosporines could be supplied from the symbiont, diet, or clam itself, and are then translocated to the cells around the epithelium layer. Once there, they may be converted to a group of mycosporines better suited to a role as sunscreen with λ_max_ of 320–330 nm, which is the wavelength that would most severely damage shallow water organisms.

UV absorption displayed two different patterns in the mantle: a strong absorption in the surface layer, up to a depth of approximately 50 μm, and a weaker absorption diffused towards much deeper regions (~500 μm) of the mantle. The outer surface of the mantle contains zooxanthellae that reside in the zooxanthellal tubular system^14^, iridophores, eyes, muscles, and a covering epithelial cell layer^18,25^. The result of MS imaging clearly supported our direct observation that the 50-μm surface layer afforded the highest UV-B absorption. Judging from the thickness of the UV-B absorption pattern, the layer of mycosporine-containing cells seemed to be much wider than a single outermost epithelial cell layer, and extended to the cells of the underlying connective tissue. The type and functions of these cells remain to be investigated. It is noteworthy that the host cells in the mantle showed little or no UV-A absorbance. Although the UV-A absorbers usujirene/palythene were localized in the outermost layer of the mantle, their concentration was very low relative to that of other mycosporines. This findings suggests that UV-A light, which may have detrimental effects on symbiont photosynthesis^12^, can be attenuated to a certain degree by a small amount of UV-A absorbing secondary mycosporines.

Although the possible role of mycosporines as sunscreen in giant clams has been suggested previously based on the increasing concentration of mycosporines towards the surface of the mantle^12^, the presently reported differential sub-tissue localizations of multiple mycosporines in the mantle was a surprise. Considering that harmful sunlight should be eliminated at the outermost level, and not at the depth inhabited by zooxanthellae, the present study provides the first empirical evidence that mycosporines indeed act as sunscreen by localizing to the very surface of *T*. *crocea*. Our observations, together with previous reports, suggest that the conversion of mycosporines by either the host or symbiont occurs widely in host-symbiont systems of coral reef ecosystems, where protection from severe sunlight is one of the greatest issues for survival. The origin of mycosporines, however, remains to be investigated. Furthermore, transformation, delivery, and dynamics of mycosporines in coral reef invertebrates are also open questions. Studies regarding the above topics are in progress in our laboratory.

## Methods

### Chemicals

LC-MS-grade solvents were used for all analyses. Standard mycosporines were isolated from various sources and the structures were determined on the basis of their spectral data from high-resolution MS, UV, and nuclear magnetic resonance (Supplementary information).

### Animal specimens

Three live specimens of *T*. *crocea* were collected in the coastal water of Ishigaki Island, Okinawa, Japan, by a local fisherman. The shell lengths of these clams were 12.5 cm (individual 1), 10.0 cm (individual 2), and 9.0 cm (individual 3). The clam specimens were kept in an outdoor open tank with running seawater for several days. The clams were dissected to separate the mantles from other visceral and adductor parts. Each mantle was cut in two parts, which were used separately for the LC-MS/MS and MS imaging analyses. Mantle samples for LC-MS/MS analysis were stored at −20 °C immediately after dissection. Mantle samples for MS imaging were cut into small pieces (ca. 10 × 10 mm), immersed in liquid N_2_, and then stored at −80 °C pending use. Specimens from the three individual clams were equally processed prior to further experiments.

### LC-MS/MS analysis

A lyophilized clam sample was weighted (~15 mg) in a 2.0-mL microtube. Clam metabolites were extracted with 50 μL/mg (dry weight) of single-phase extraction solvent (water:methanol:chloroform = 2:5:2, v/v/v; containing 1 μg/mL HEPES as an internal standard) using an ultrasonic homogenizer (Smurt NR 50 M; Microtec, Funabashi, Chiba, Japan) for 2 min on ice. After vortexing, the sample was centrifuged at 4 °C and 16,000 × *g* for 3 min. The extract was transferred to a new 1.5-mL microtube, water was added (4/9 volumes of transferred extracts) to adjust the final solvent composition (water:methanol:chloroform = 6:5:2, v/v/v), vortexed again, and centrifuged at 4 °C and 16,000 × *g* for 3 min to separate the upper and lower phases. The upper polar phase was transferred to a new 1.5-mL vial and dried using a centrifugal vacuum evaporator. The polar extracts were resuspended and diluted two-fold with water, subjected to UV-HPLC analysis, diluted (1:100) in new 1.5-mL vials, and used for HPLC-MS/MS analysis. The samples were prepared in triplicate.

UV-HPLC was carried out using a PDA detector with ChromNAV ver.2 software (JASCO, Tokyo, Japan). Mycosporines were separated with solvent A (water with 0.05% trifluoroacetate) and solvent B (methanol with 0.05% trifluoroacetate) according to the following gradient: 0–2 min 2% B, 2–15 min ramp to 50% B, 15–17 min 50% B, 17–18 min ramp to 2% B, 18–26 min 2% B). A C18 column (Inertsil ODS-3V, 4.6 mm × 150 mm; GL Science, Tokyo, Japan) was used. The flow rate was set to 1.2 mL/min and the column oven was set to 35 °C. The injection volume of the samples was 25 μL.

HPLC-MS/MS was performed on a quadrupole TOF mass spectrometer (TripleTOF 5600+; AB Sciex, Framingham, MA, USA). A C18 column (HSS-T3, S-3.5 μm, 2.1 mm × 150 mm, Waters, Milford, MA, USA) was used for LC separation with solvent A (water with 0.1% formic acid) and solvent B (methanol with 0.1% formic acid) according to the following gradient: 0–2 min 1% B, 2–10 min ramp to 20% B, 10–18 min ramp to 99% B, 18–23 min 99% B, 23–24 min ramp to 1% B, 24–30 min 1% B. The flow rate was set to 0.2 mL/min, the column oven was set to 35 °C, and 5 μL of sample was injected. MS data were collected in scan mode in the range *m*/*z* 60–2000 in positive ion mode. MS/MS analysis was performed in high-resolution mode with information-dependent acquisition. MS/MS data were acquired in the MS range *m*/*z* 20–1200 with collision energy of +20–50 eV.

### Mass data analysis

LC-MS data were processed using PeakView 2.1 and MasterView 1.0 software (AB Sciex) with metabolites identification based on our in-house metabolite library (MS and retention time). PCA analysis was performed using MarkerView 1.2.1 software (AB Sciex). Peak areas of mycosporines were compared on an R 3.6.0 platform.

### MS imaging

Consecutive 10-μm sections were cut directly from frozen samples using a cryostat (CM 1950; Leica Microsystems, Wetzlar, Germany). The serial sections were mounted onto glass slides for HE staining and onto indium tin oxide-coated glass slides (Bruker Daltonics, Billerica, MA, USA) for MS imaging. After MS imaging, the sections were also subjected to HE staining for morphological observation. Samples were prepared as described previously^26,27^. Briefly, a matrix solution containing 50 mg/mL 2,5-dihydroxybenzoic acid in methanol:water (8:2, v/v) was used, with 1–2 mL of it prepared before use and sprayed uniformly over the frozen sections using an airbrush with a 0.2-mm nozzle (Procon Boy FWA Platinum; Mr. Hobby, Tokyo, Japan). MS imaging analysis was performed using TOF/TOF 5800 (AB Sciex) and SolariX, FT-ICR (Bruker Daltonics) mass spectrometers. To optimize FT-ICR MS, we set the lock mass to *m*/*z* 200–500, and the spatial resolution to 150 μm for the mass imaging dataset.

### UV light photography

Mantle tissue samples of 10 μm thickness prepared as above were fixed in 4% paraformaldehyde for 24 h, transferred to sucrose gradients, and stored in 30% sucrose. Frozen sections were stained with HE, and observed under a Nikon microscope. A number of 30 μm-thick slices of mantle tissue prepared as described above, were placed on a glass slide and dried. The sections were subjected to UV macro- or micro-photography according to a previously reported method^28^ except for using a digital camera instead of a film camera. A UV-B emitting fluorescent tube (100 V 6 W, GL6E; Sankyo Denki, Hiratsuka, Japan) was used as a light source. A NEX-5N camera (Sony, Tokyo, Japan) without UV protection filter was used with a quartz lens (f = 25 mm, F2.8, FL-BC2528; Ricoh, Tokyo, Japan) or a Nikon Fluor 10× objective lens (NA = 0.5, 160/0.17) for photographic recording. UV band-pass filters for 320 ± 10, 337 ± 10, and 365 ± 10 nm (central wavelength ± 50% transparent band-pass wavelength: Edmund Optics Inc., Barrington, NJ, USA) were used. The camera and lens equipped with macro-extension bellows or with a macro-helicoid extension ring were set on a macrophoto-stand. The filter was inserted between the lens and the bellows or placed in front of the lens.

### Transmission electron microscopy

To observe the fine structure of the *T*. *crocea* mantle, we prepared an additional clam sample different from the above mentioned three clams. An edge of the mantle of *T*. *crocea* was fixed with 2.5% glutaraldehyde in seawater and post-fixed with 2% OsO_4_ in seawater, then processed and observed as described previously^29^.

## Supplementary information

Supplementary Information.
