# Supplementary material for: Mass spectrometry imaging reveals differential localization of natural sunscreens in the mantle of the giant clam Tridacna crocea
Source: Sci Rep. 2020 Jan 20;10:656. doi: 10.1038/s41598-019-57296-9 (PMC6971080; doi:10.1038/s41598-019-57296-9)
Supplement: Supplementary file 1 — Supplementary Information. [file 41598_2019_57296_MOESM1_ESM.pdf]

Supplementary Information

Mass spectrometry imaging reveals differential localization of natural sunscreens in the mantle of the giant clam *Tridacna crocea*

Naoko Goto-Inoue[1], Tomohiko Sato[1], Mizuki Morisasa[1], Hiroshi Yamashita[2], Tadashi Maruyama[3], Hiroki Ikeda[4], and Ryuichi Sakai[4*]

[1]Department of Marine Science and Resources, College of Bioresource Sciences, Nihon University, 1866 Kameino, Fujisawa, Kanagawa 252-0880, Japan
[2]Research Center for Subtropical Fisheries, Seikai National Fisheries Research Institute, Japan Fisheries Research and Education Agency, Ishigaki, Okinawa 907-0451, Japan
[3]School of Marine Biosciences, Kitasato University, 1-15-1, Kitazato, Minami, Sagamihara, Kanagawa 252-0374, Japan
[4]Faculty and Graduate School of Fisheries Sciences, Hokkaido University, 3-1-1 Minato-cho, Hakodate, 041-8611, Japan

***Corresponding author:** Ryuichi Sakai

Tel: +81-138-40-5552, E-mail: ryu.sakai@fish.hokudai.ac.jp

Table of Contents

Figure 1S. Principle component analysis of extracts from EL and IL. A Score Plot. Score plots for three specimen from EL and IL were shown. 3

Figure 2S. Principle component analysis of extracts from EL and IL. A Loading Plot. ▲precursor and progenitor; ■primary; ●secondary mycosporines. 4

Table 1S. List of metabolites identified in the target analysis by LC-MS experiment. 5

Figure 3S. Peak retention time (tR) and UV absorption for standard mycosporines on PDA chromatogram. 7

Figure 4S. UV absorption and retention time for the annotated mycosporine peaks in Fig. 1. 8

Figure 5S. FT-ICR mass spectra of three representative mycosporines. The ion intensities were compared between EL and IL. 9

Spectral data of Standard Mycosporines 10

- **4-deoxygadusol** 11
- **Mycosporine-Gly** 13
- **Shinorine** 15
- **Palythinol** 17
- **Asterina-330** 19
- **Porphyla-334** 21
- **Palythenic acid (Z)** 23
- **Usujirene/palythene 7:3 mixture** 25
- **Mycosporine-2-glycine** 29
- **Palythine** 31

References 32

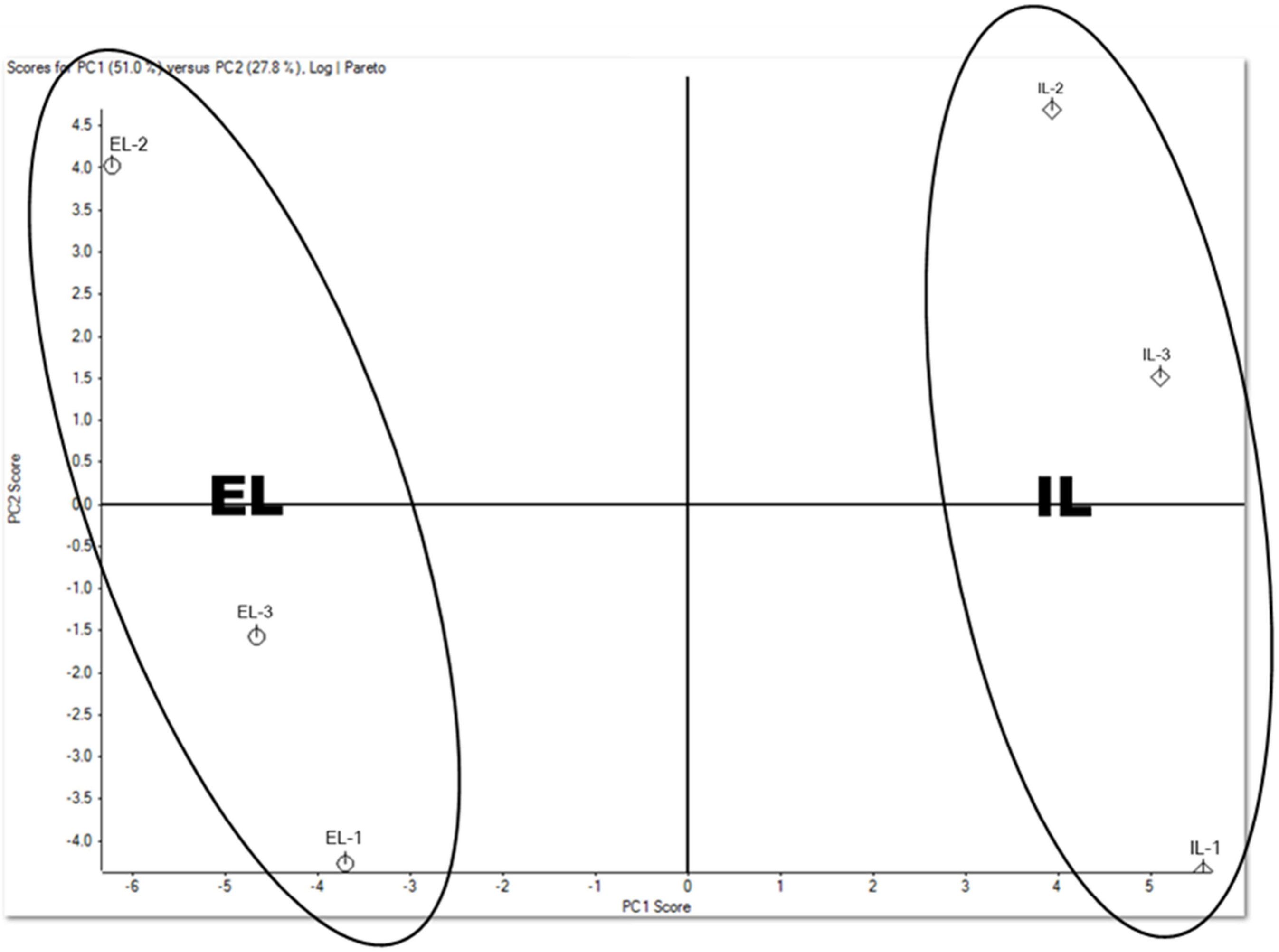

Figure 1S. Principle component analysis of extracts from EL and IL. A Score Plot. Score plots for three specimen from EL and IL were shown.

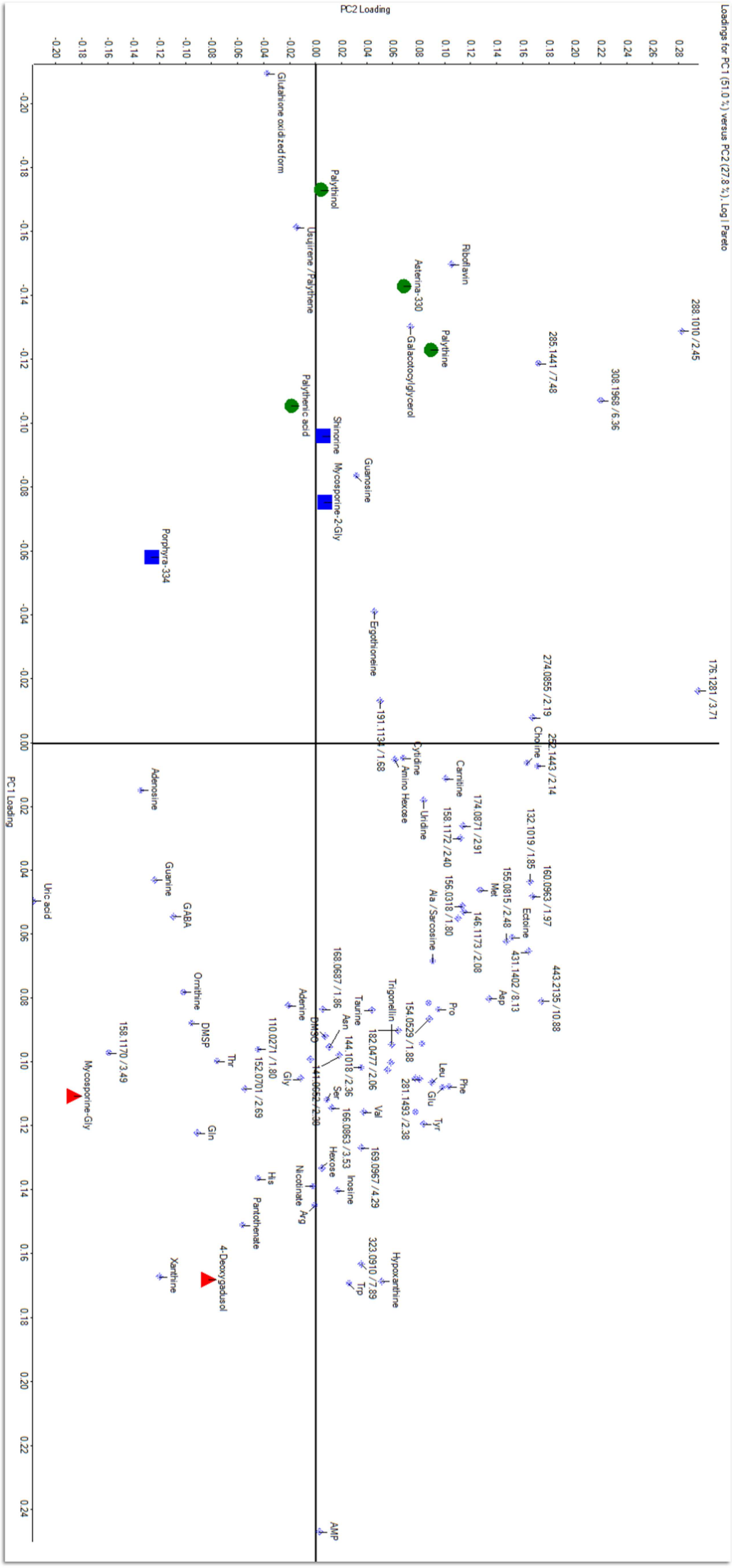

Figure 2S. Principle component analysis of extracts from EL and IL. A Loading Plot. ▲precursor and progenitor; ■primary; ●secondary mycosporines.

Table 1S. List of metabolites identified in the target analysis by LC-MS experiment.

Peak Name	Extracted m/z	Molecular Formula	$[M+H]^+$	Mass (Da)	Ret. Time min	Group	Identification
Gly	76.0393	C2H5NO2	76.03930	75.03203	1.75	Amino acids	STD
DMSO	79.0212	C2H6OS	79.02121	78.01394	2.97	Others	STD
Ala / Sarcosine	90.055	C3H7NO2	90.05495	89.04768	1.82	Amino acids	STD
GABA	104.0706	C4H9NO2	104.07060	103.06333	1.91	Amino acid deriv.	STD
Ser	106.0499	C3H7NO3	106.04987	105.04259	1.76	Amino acids	STD
Pro	116.0706	C5H9NO2	116.07060	115.06333	2.16	Amino acids	STD
Glycine betaine	118.0863	C5H11NO2	118.0813	117.07898	1.93	Amino acid deriv.	STD
Val	118.0863	C5H11NO2	118.0813	117.07898	3.01	Amino acids	STD
Thr	120.0655	C4H9NO3	120.06552	119.05824	1.86	Amino acids	STD
Nicotinate	124.0393	C6H5NO2	124.03930	123.03203	3.88	Amino acid deriv.	STD
Taurine	126.0219	C2H7NO3S	126.02194	125.01467	1.78	Amino acid deriv.	STD
Leu / Ile	132.1019	C6H13NO2	132.10190	131.09463	6.57	Amino acids	STD
Asn	133.0608	C4H8N2O3	133.06077	132.05349	1.77	Amino acids	STD
Asp	134.0448	C4H7NO4	134.04478	133.03751	1.79	Amino acids	STD
Adenine	136.0618	C5H5N5	136.06177	135.0545	3.57	Nucleic acids	STD
Hypoxanthine	137.0458	C5H4N4O	137.04579	136.03851	5.15	Nucleic acids	STD
Trigonelline	138.055	C7H7NO2	138.0500	137.04768	2.16	Amino acid deriv.	STD
Ectoine	143.0815	C6H10N2O2	143.08150	142.07423	2.24	Amino acid deriv.	STD
Gln	147.0764	C5H10N2O3	147.07642	146.06914	1.82	Amino acids	STD
Lys	147.1128	C6H14N2O2	147.11280	146.10553	1.6	Amino acids	STD
Glu	148.0604	C5H9NO4	148.06043	147.05316	1.91	Amino acids	STD
Met	150.0583	C5H11NO2S	150.05833	149.05105	3.68	Amino acids	STD
Guanine	152.0567	C5H5N5O	152.05668	151.04941	3.66	Nucleic acids	STD
Xanthine	153.0407	C5H4N4O2	153.0470	152.03343	6.23	Nucleic acids	STD
His	156.0768	C6H9N3O2	156.0718	155.06948	1.69	Amino acids	STD
Carnitine	162.1125	C7H15NO3	162.11247	161.10519	2.16	Amino acid deriv.	STD
Phe	166.0863	C9H11NO2	166.0813	165.07898	10.09	Amino acids	STD
Arg	175.119	C6H14N4O2	175.1140	174.11168	1.73	Amino acids	STD

Amino Hexose	180.0866	C6H13NO5	180.0817	179.07937	1.9	Sugars	STD
Hexose	181.0707	C6H12O6	181.07066	180.06339	1.76	Sugars	STD
Tyr	182.0812	C9H11NO3	182.08117	181.07389	6.6	Amino acids	STD
Trp	205.0972	C11H12N2O2	205.09715	204.08988	13.46	Amino acids	STD
Pantothenate	220.1179	C9H17NO5	220.11795	219.11067	11.77	Others	STD
Ergothioneine	230.0958	C9H15N3O2S	230.09577	229.0885	2.44	Amino acid deriv.	STD
Adenosine	268.104	C10H13N5O4	268.10403	267.09675	8.39	Nucleic acids	STD
Guanosine	284.0989	C10H13N5O5	284.09894	283.09167	8.84	Nucleic acids	STD
AMP	348.0704	C10H14N5O7P	348.07036	347.06309	4.22	Nucleic acids	STD
Riboflavin	377.1456	C17H20N4O6	377.14556	376.13828	15.47	Others	STD
Glutathione oxidized form	613.1592	C20H32N6O12S2	613.15924	612.15196	7.31	Amino acid deriv.	STD
Ornithine	133.0972	C5H12N2O2	133.09715	132.08988	1.6	Amino acid deriv.	STD
Uric acid	169.0356	C5H4N4O3	169.03562	168.02834	4.79	Others	STD
Cytidine	244.0928	C9H13N3O5	244.0928	243.08552	3.73	Nucleic acids	STD
Galactosyl glycerol	255.1074	C9H18O8	255.10744	254.10017	2.26	Sugars	NMR
Choline	104.107	C5H14NO		104.10754	1.81	Others	STD
4-Deoxygadusol	189.0758	C8H12O5	189.07575	188.06847	6.16	MAAs	NMR
DMSP	135.0472	C5H10O2S	135.04743	134.04015	1.84	Amino acid deriv.	STD
Uridine	245.0768	C9H12N2O6	245.07681	244.06954	6.67	Nucleic acids	STD
Palythine	245.1132	C10H16N2O5	245.1132	244.10592	2.65	MAAs	NMR
Mycosporine-Gly	246.0972	C10H15NO6	246.09721	245.08994	5.82	MAAs	NMR
Inosine	269.088	C10H12N4O5	269.08804	268.08077	8.77	Nucleic acids	STD
Usujirene / Palythene	285.1445	C13H20N2O5	285.1445	284.13722	9.72	MAAs	NMR
Asterina-330	289.1394	C12H20N2O6	289.13941	288.13214	3.02	MAAs	NMR
Mycosporine-2-Gly	303.1187	C12H18N2O7	303.11868	302.1114	2.73	MAAs	NMR
Palythinol	303.1551	C13H22N2O6	303.15506	302.14779	4.92	MAAs	NMR
Palythenic acid	329.1343	C14H20N2O7	329.13433	328.12705	6.5	MAAs	NMR
Shinorine	333.1292	C13H20N2O8	333.12924	332.12197	2.53	MAAs	NMR
Porphyra-334	347.1449	C14H22N2O8	347.14489	346.13762	4.63	MAAs	NMR

STD: commercial standard compound.

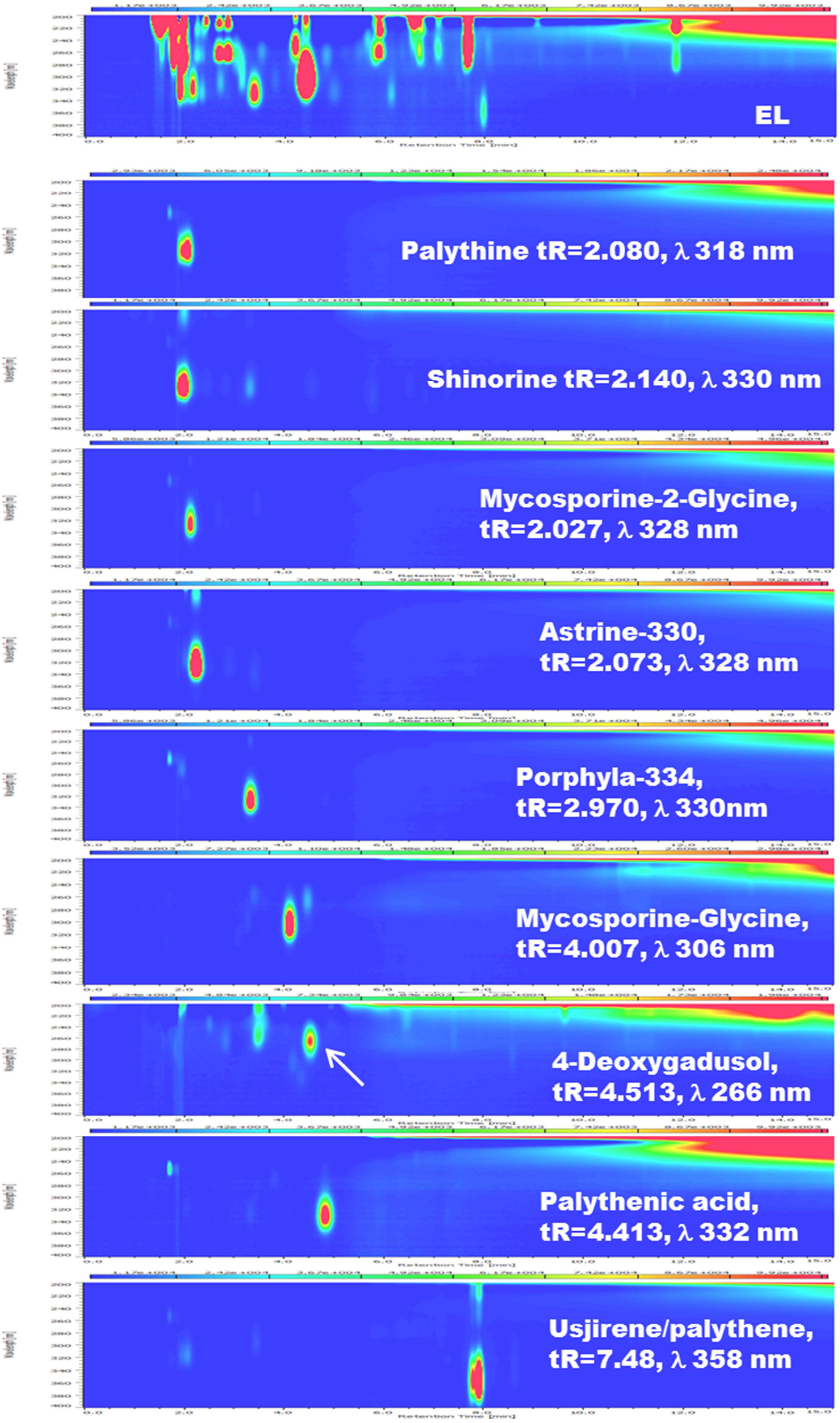

Figure 3S. **Peak retention time (tR) and UV absorption for standard mycosporines on PDA chromatogram.**

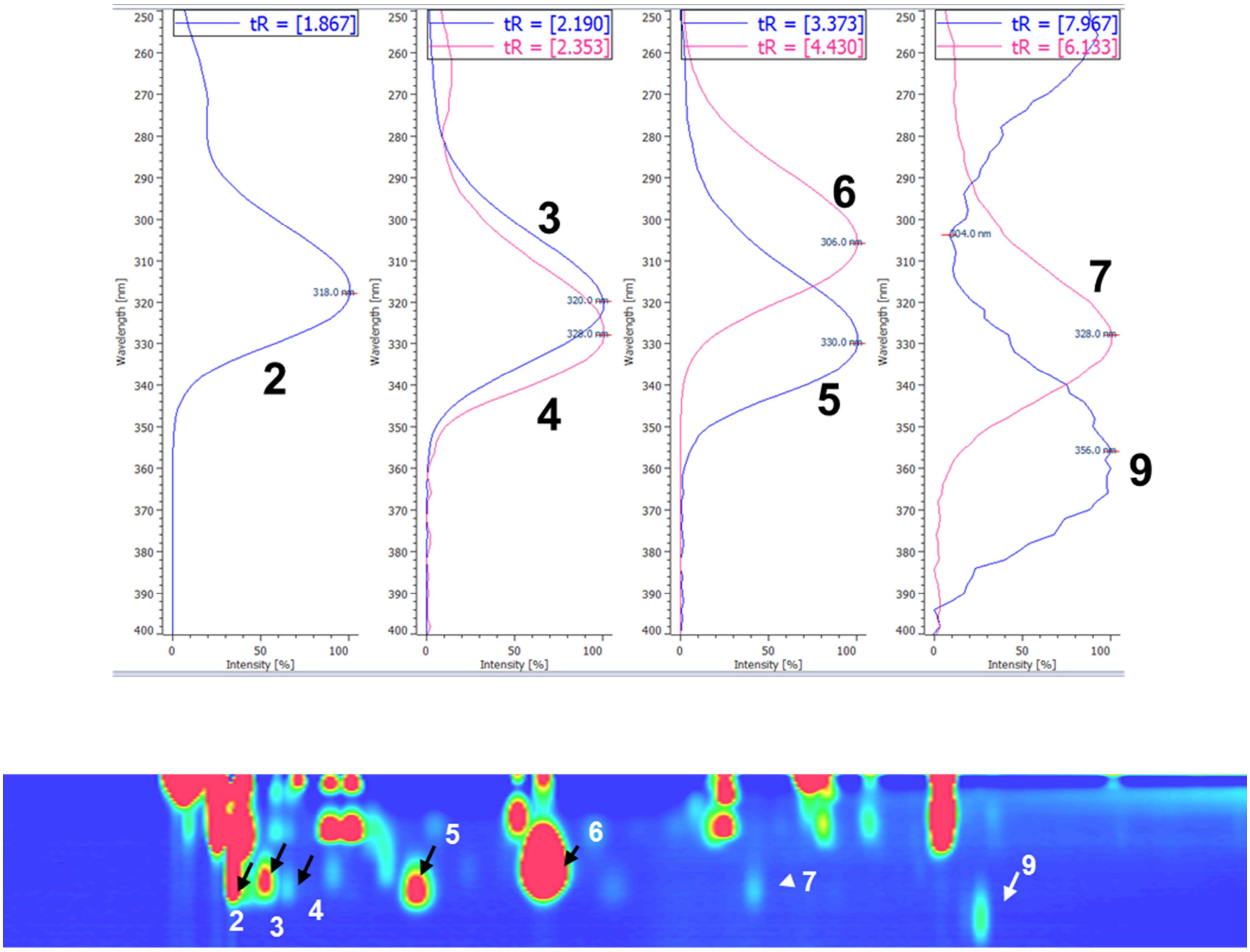

Figure 4S. UV absorption and retention time for the annotated mycosporine peaks in Fig. 1.

Peak annotation: 2) palythine, 3) unidentified mycosporine A, 4) asterina-330, 5) porphyra-334, 6) mycosporine-Gly, 7) unidentified mycosporine B, 8) inosine, 9) usujirene/palythene.

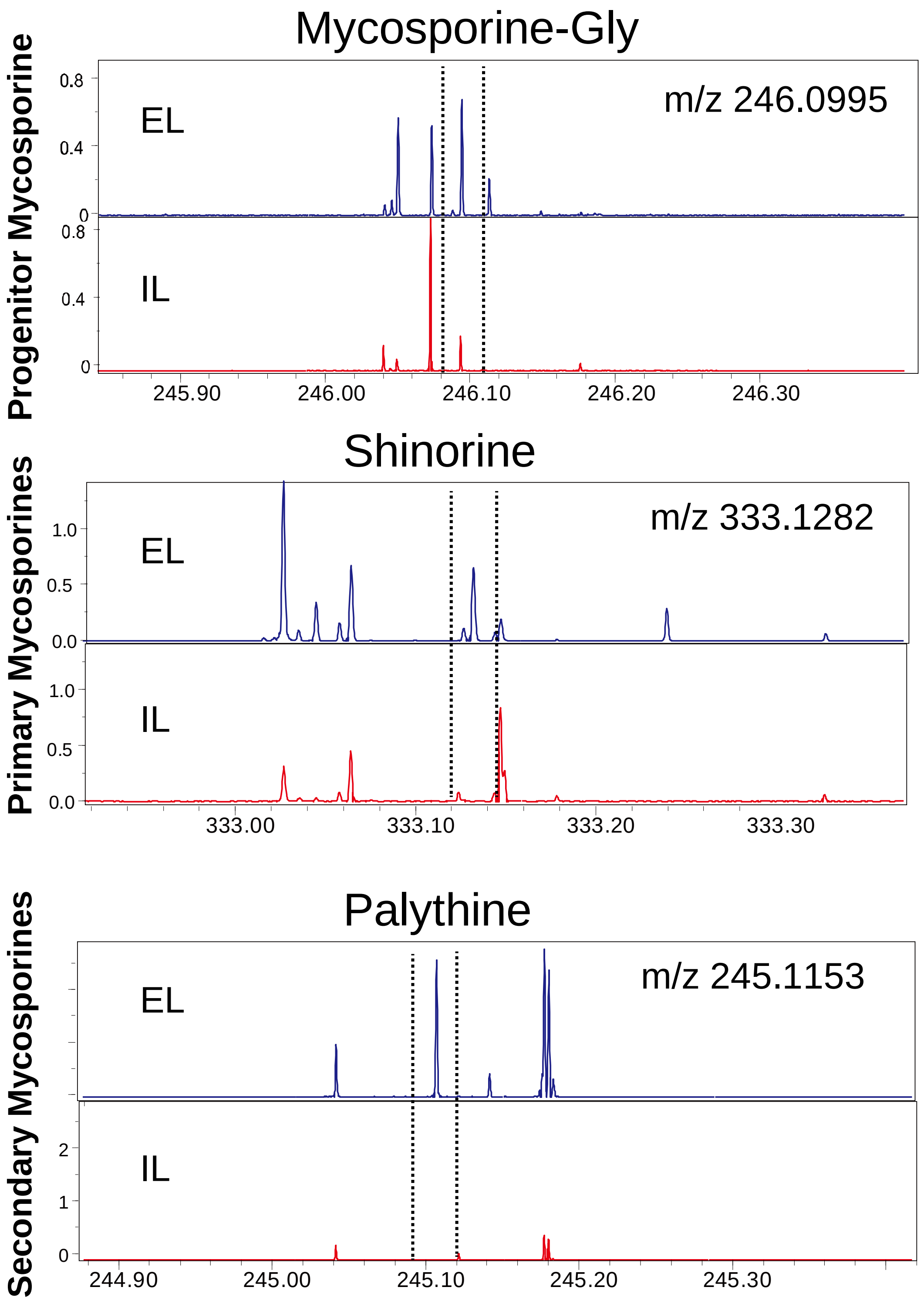

Figure 5S. FT-ICR mass spectra of three representative mycosporines. The ion intensities were compared between EL and IL.

Spectral data of Standard Mycosporines

^{1}H NMR (400 MHz, D_2O = δ 4.65, at 25 ℃) and HIRES ESIMS and MS/MS data for 4-deoxygadusole and mycosporines used in this study are listed below.

4-deoxygadusol

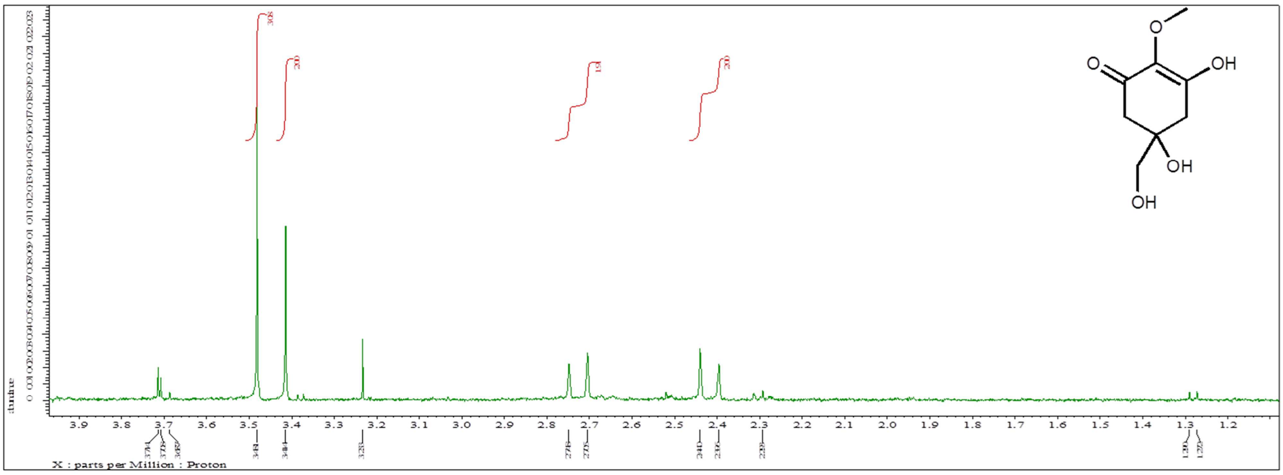

4-Deoxygadusol in D_2O		reported values[1]	
position	δH , type (J in Hz)	δH , type (J in Hz)	δH (⊿)
CH_2-4,6	2.42, d, 2H (17.7)	2.60, d, 2H (17)	-0.18
	2.73, d, 2H (17.4)	2.90, d, 2H (17)	-0.17
CH_2-7	3.41, s, 2H	3.57, s, 2H	-0.16
CH_3-8	3.48, s, 3H	3.64, s, 3H	-0.16

4-deoxygadusol

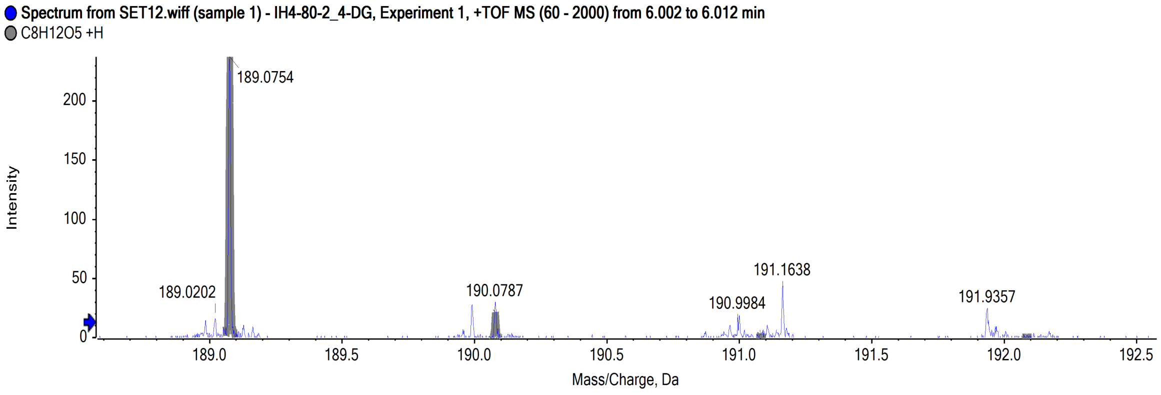

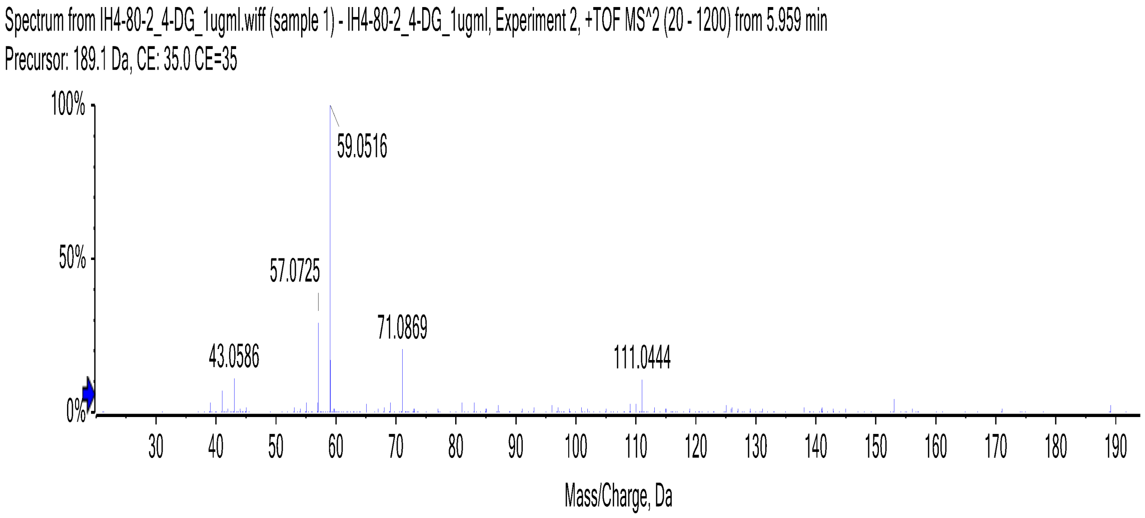

HRESIMS data (upper) with molecular ion (MH^+) calcd for $C_8H_{13}O_5$, 189.0763; found 189.0756, and MS/MS data (lower) for 4-deoxygadusol.

Mycosporine-Gly

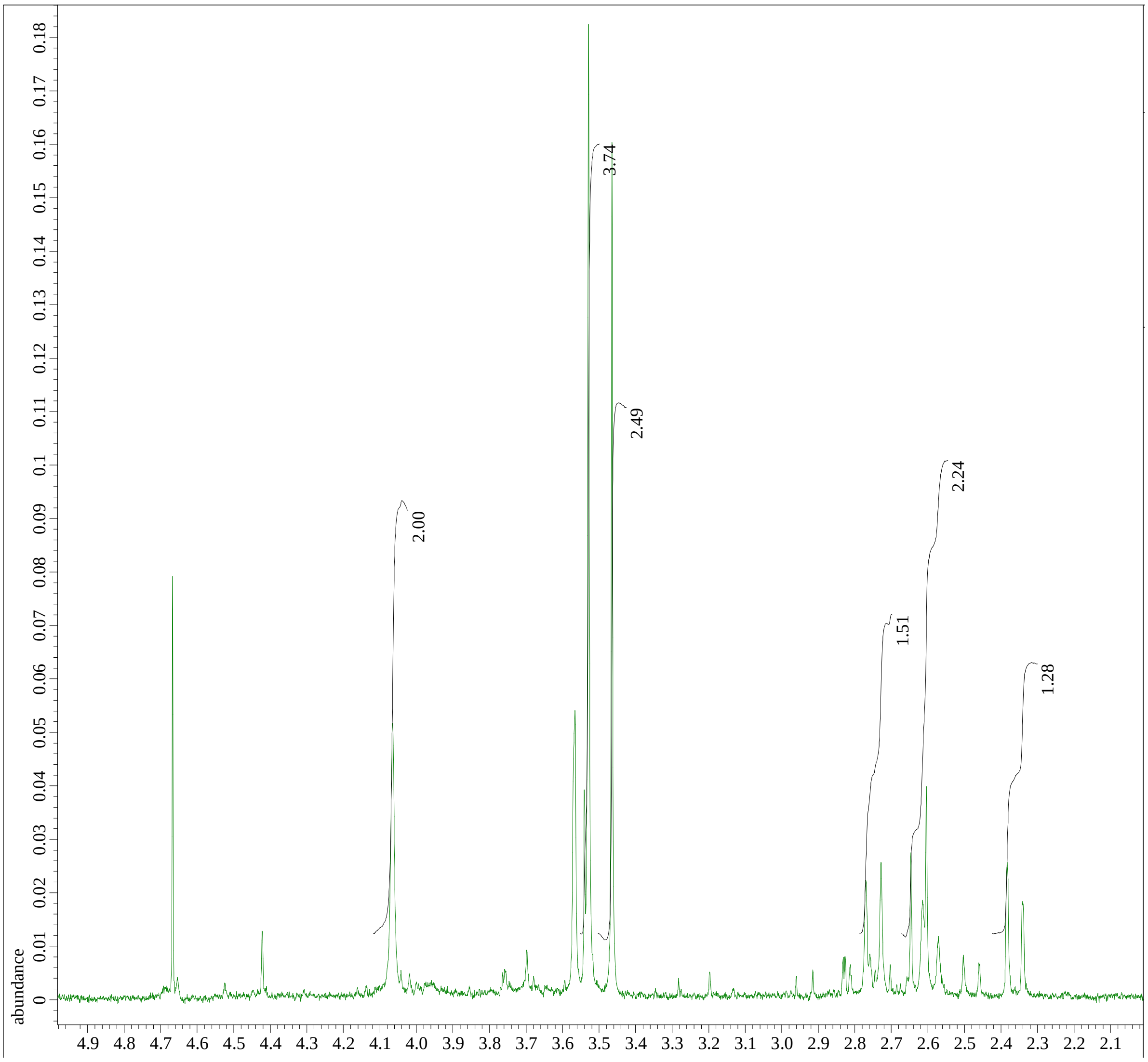

Mycosporine-Gly in D_2O		Reported values (D_2O)[1]	
position	δH , type (J in Hz)	δH , type (J in Hz)	δH (⊿)
CH_2-4*	2.35-2.65 ABq (17)	2.50-2.72 ABq (17)	-0.15~0.07
CH_2-6*	2.58-2.75 ABq (17)	2.72-2.83 ABq (17)	-0.13~0.08
CH_2-7	3.45, s, 2H	3.57, s, 2H	-0.12
CH_3-8	3.51, s, 3H	3.64, s, 3H	-0.13
CH_2-9	4.05, s, 2H	4.24, s, 2H	-0.19

*Assignments may be interchanged.

Mycosporine-Gly

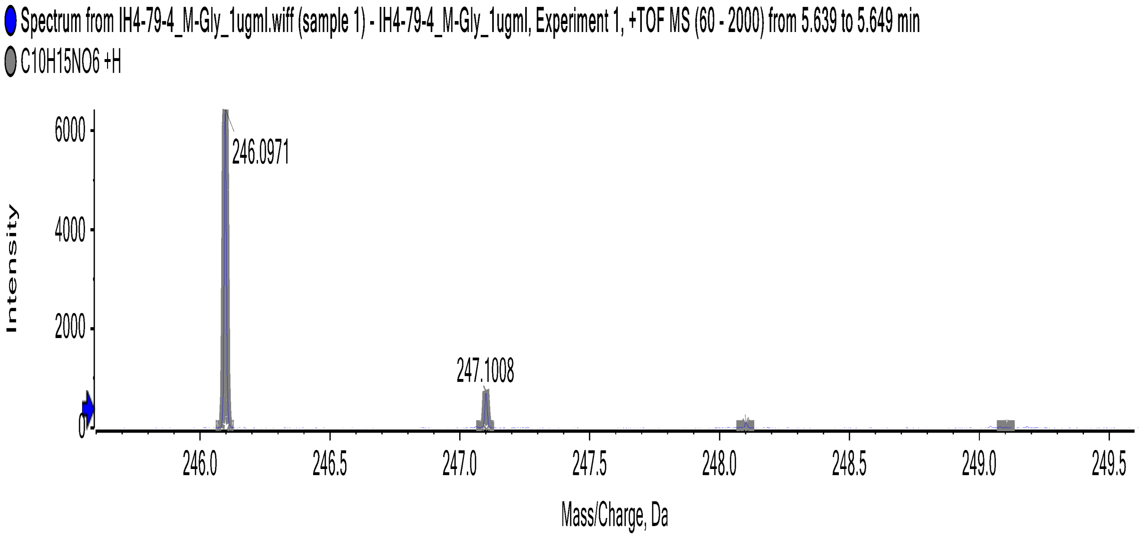

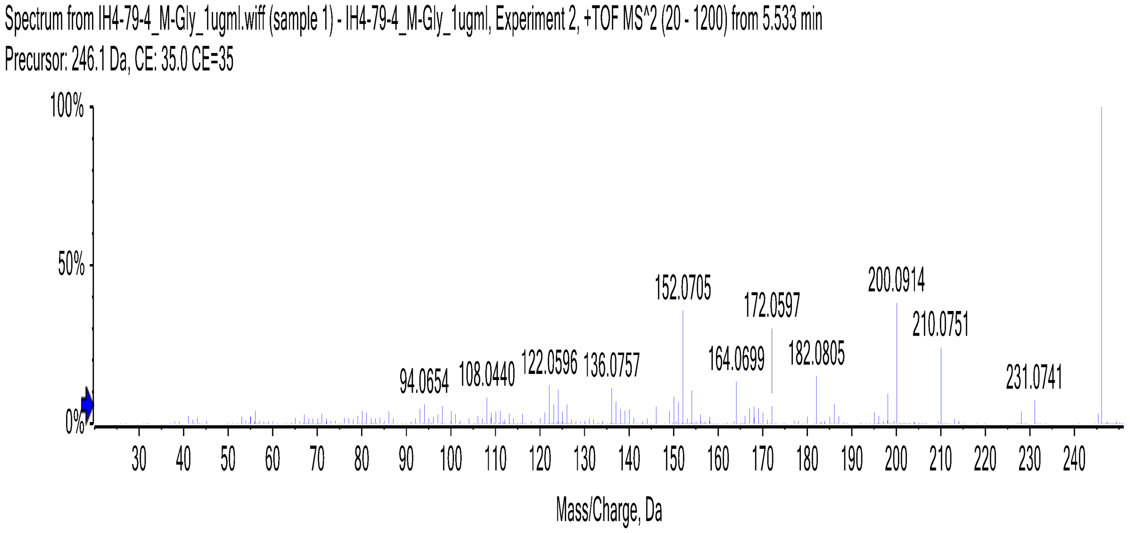

HRESIMS data (upper) with molecular ion (MH^+) calcd for $C_{10}H_{16}NO_6$, 246.0978; found 246.0971, and MS/MS data (lower) for mycosporine-glycine.

Shinorine

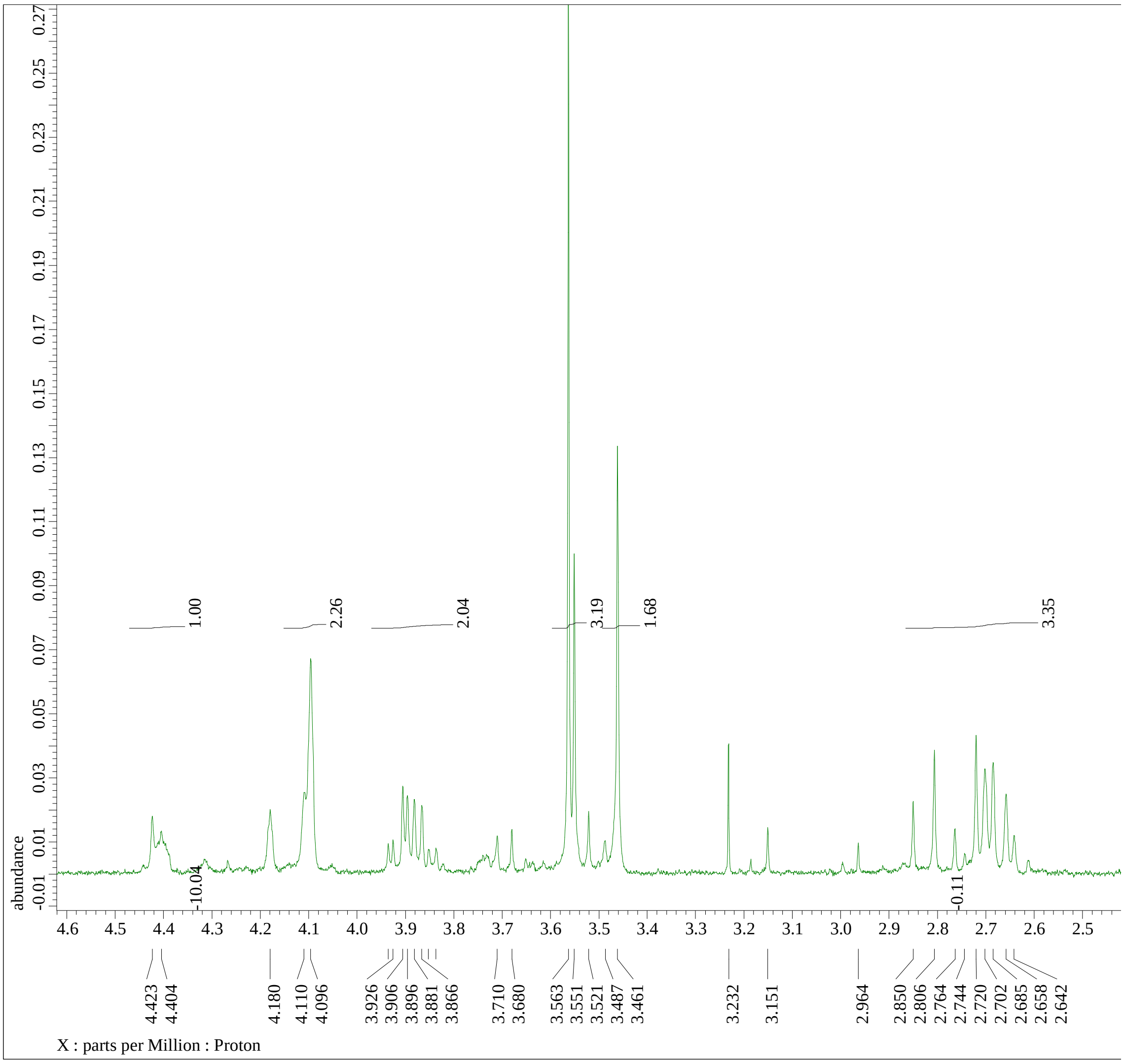

Shinorine in D_2O		Reported values[2]	
position	δH , type (J in Hz)	δH , type (J in Hz)	δH (⊿)
CH_2-4*	2.68,2.83, ABq, 2H, (17.6 Hz)	2.78, m, 2H	
CH_2-6*	2.67-2.74, ABq, 2H(17.6 Hz)	2.87, m, 2H	
CH_2-7	3.46, s, 2H	3.56, s, 2H	-0.1
CH_3-8	3.56, s, 3H	3.66, s, 3H	-0.1
CH_2-9	4.10, s, 2H	4.09, s, 2H	0.01
CH-11	4.41, m, 1H	4.48, m, 1H	-0.07
CH_2-13	3.86, dd, 1H(6.0, 11.6 Hz)<br>3.91, dd, 1H (3.6, 11.6 Hz)	3.85, dd, 2H (6.7, 13 Hz)	0.04

*Assignments may be interchanged.

Shinorine

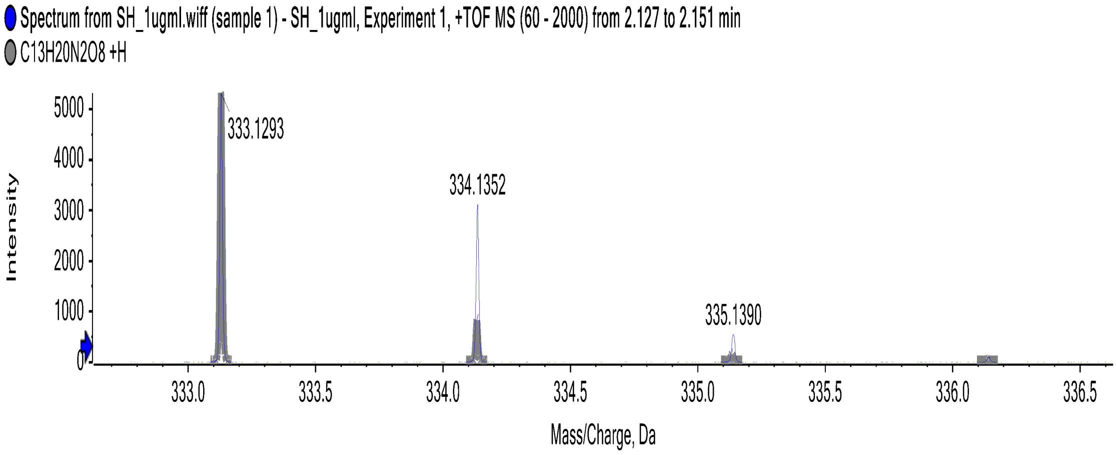

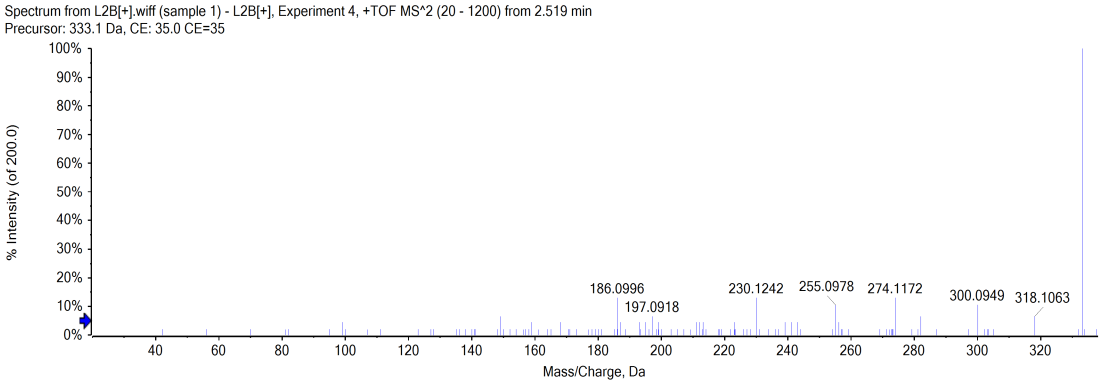

HRESIMS data (upper) with molecular ion (MH^+) calcd for $C_{13}H_{21}N_2O_8$, 333.1298; found 333.1293, and MS/MS data (lower) for shinorine.

Palythinol

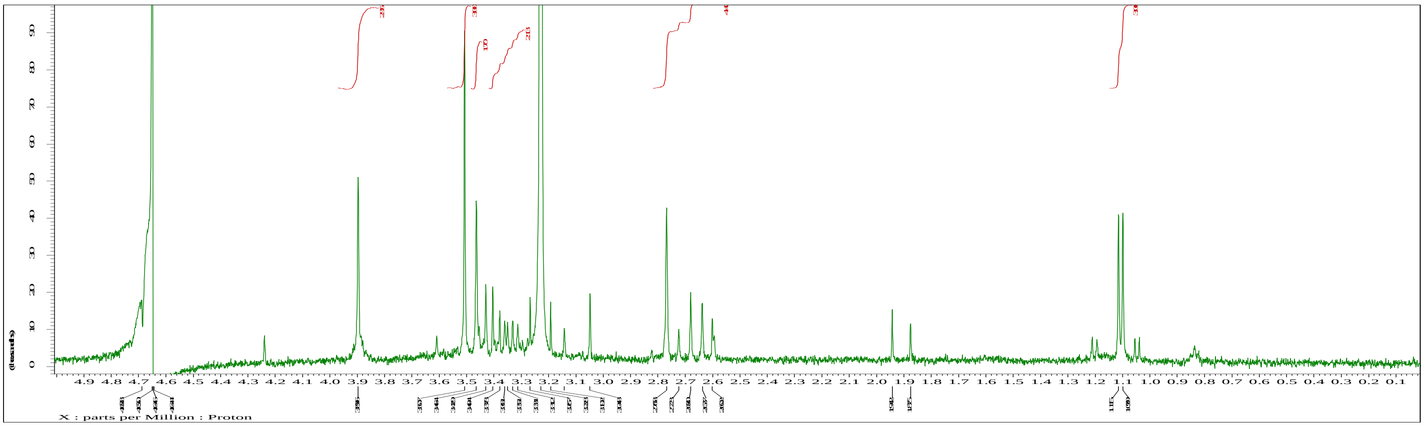

Palythinol in D_2O		Reported values[3]	
position	δH , type (J in Hz)	δH , type (J in Hz)	δH (⊿)
CH_2-4*	2.65, 2H, Abq, 17.6	2.82, 2H, Abq, 17.6	-0.17
CH_2-6*	2.77, 2H, s	2.94, 2H, Abq, 17.6	-0.17
CH_2-7	3.46, s, 2H	3.62, s, 2H	-0.16
CH_3-8	3.50, s, 3H	3.66, s, 3H	-0.16
CH_2-9	3.9 m	not annotated	
CH-11	3.9 m**	4.06 1H, m	-0.16
CH-12	1.11, 3H, d, 4.8Hz	1.26, 3H, d, 5Hz	-0.15
CH_3-13	3.3-3.4. 2H, m	3.46-3.52, 2H, m	-0.16

*Assignments may be interchanged.

**COSY correlation peaks were found between CH_3-12 and CH_2-13.

Palythinol

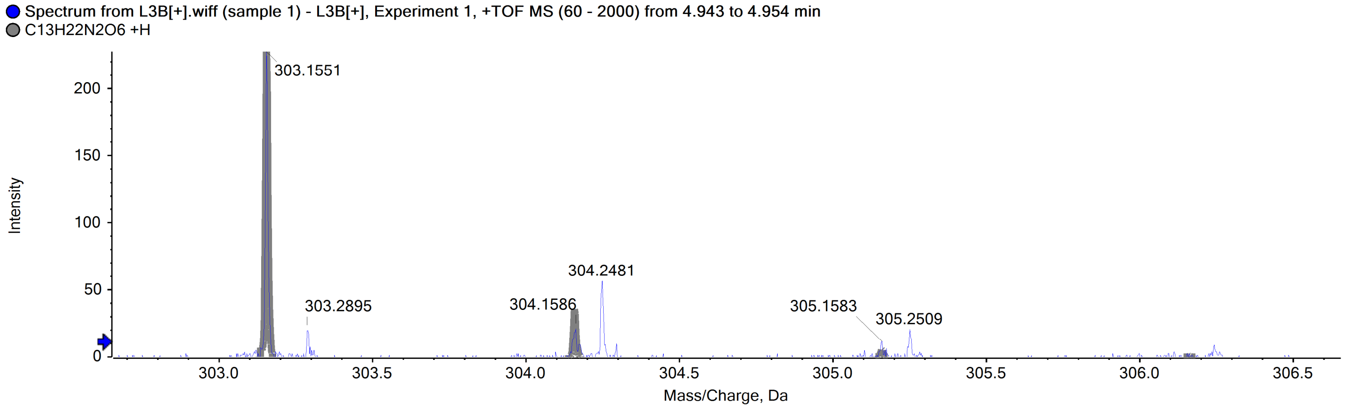

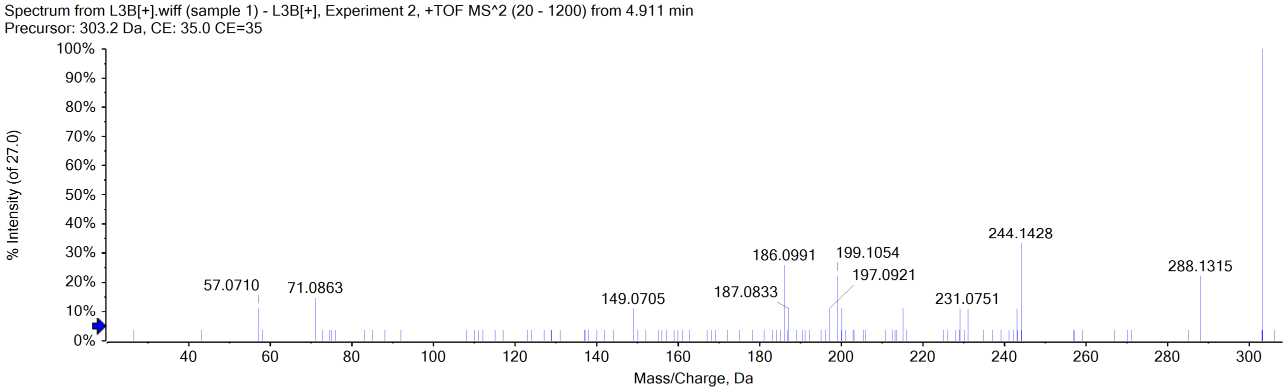

HRESIMS data (upper) with molecular ion (MH^+) calcd for $C_{13}H_{23}N_2O_6$, 303.1556; found 303.1551, and MS/MS data (lower) for palythenol.

Asterina-330

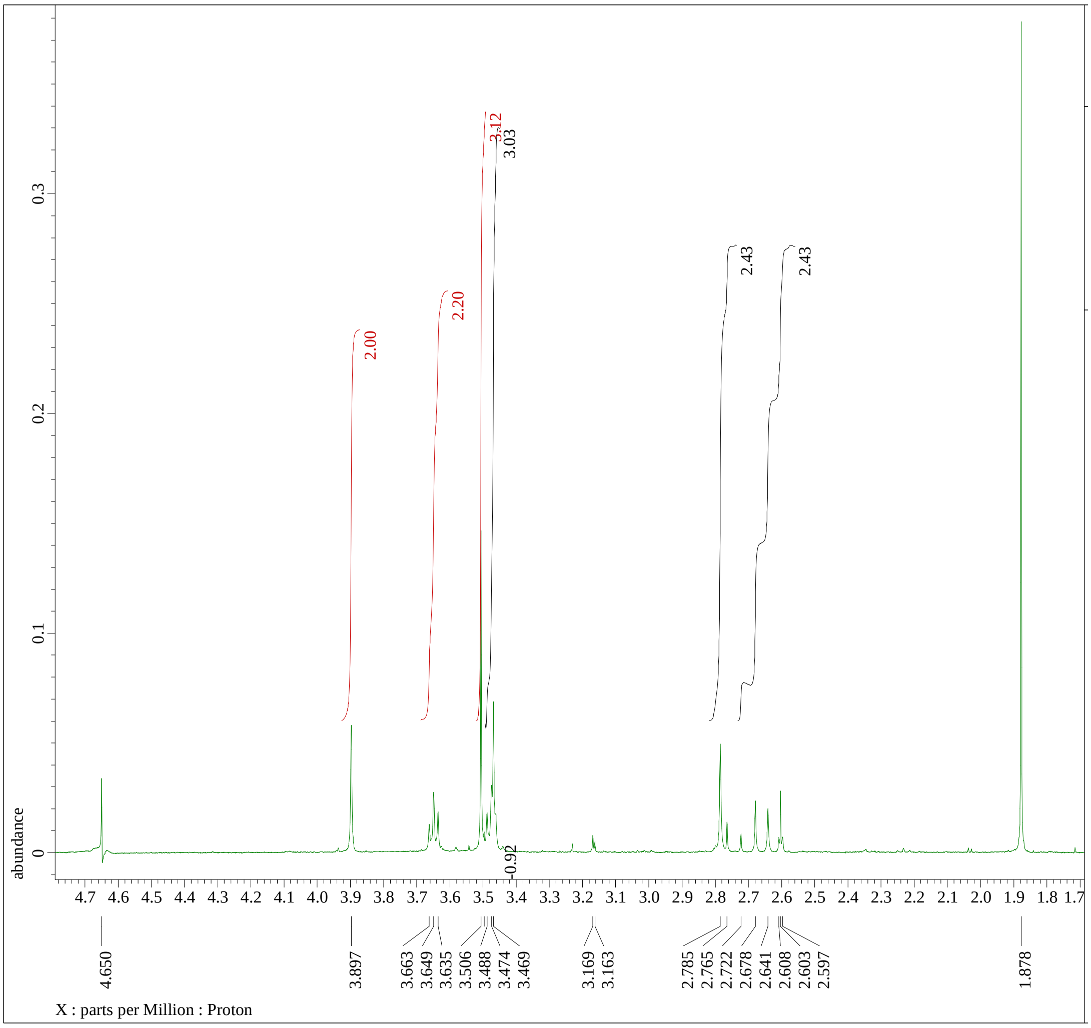

Asterina-330 in D_2O		Reported values[4]	
position	δH , type (J in Hz)		δH (⊿)
CH_2-4*	2.73, 2.84, ABq, 2H	2.63, 2.70, ABq, 2H	~-0.13
CH_2-6*	2.92, s, 2H	2.79, s, 2H	-0.13
CH_2-7	3.47, s, 2H	3.58, s, 2H	-0.11
CH_3-8	3.50, s, 3H	3.65, s, 3H	-0.15
CH_2-9	3.90, s, 2H	4.27, s, 2H	-0.37
CH_2-11	3.47, t, 2H	3.60, t, 2H	-0.13
CH_2-12	3.65, t, 2H (5.5 Hz)	3.77, t, 2H	-0.12

*Assignments may be interchanged.

Asterina-330

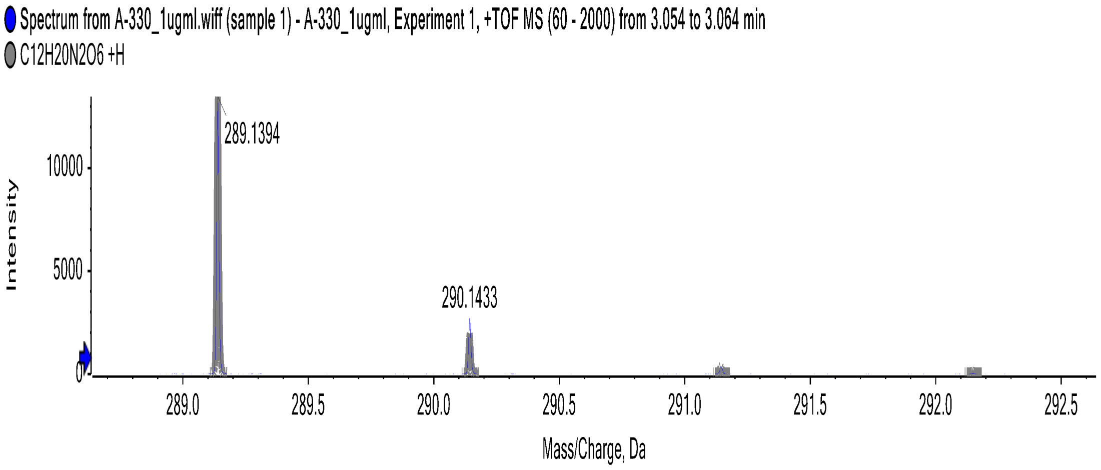

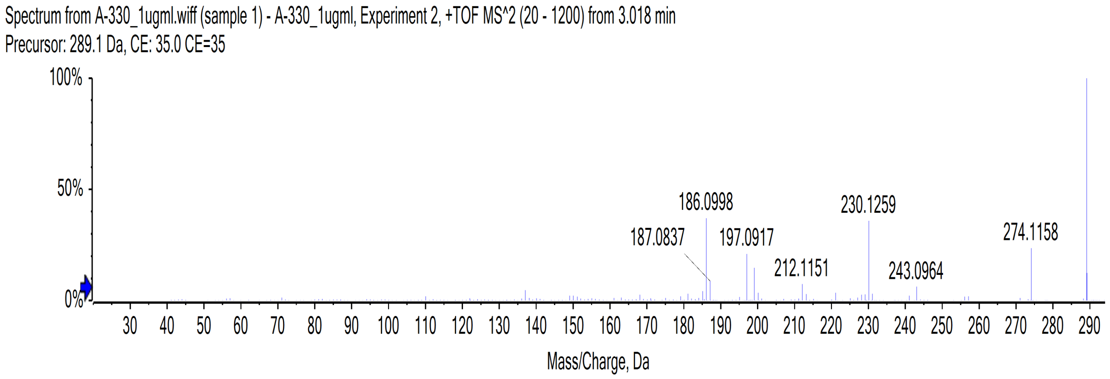

HRESIMS data (upper) with molecular ion (MH^+) calcd for $C_{12}H_{21}N_2O_6$, 289.1400; found 289.1394, and MS/MS data (lower) for Asterina-330.

Porphyla-334

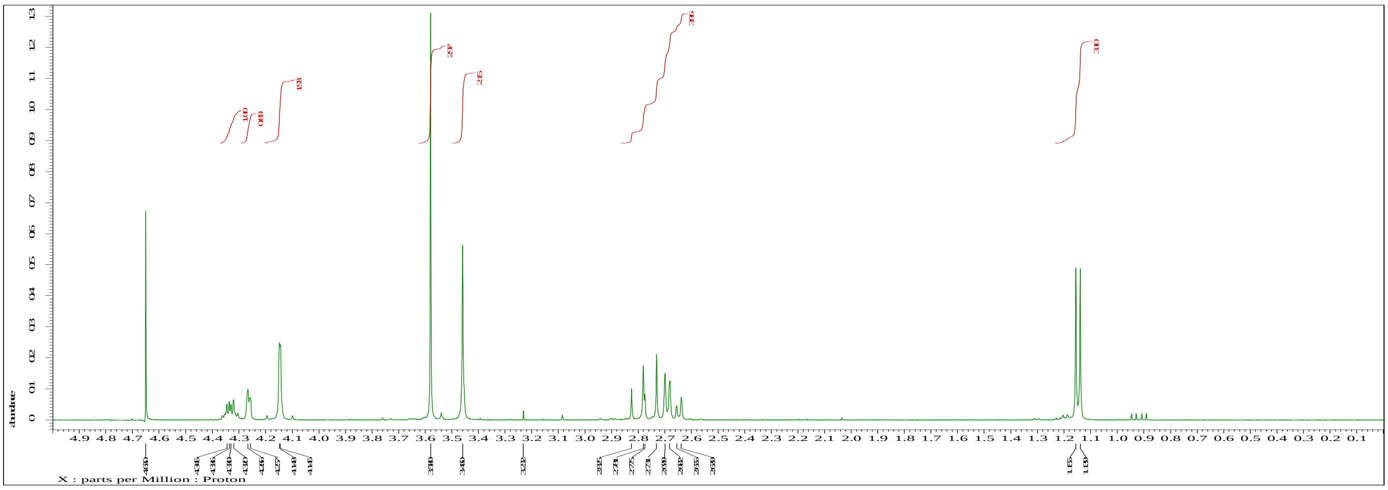

Porphyra-334 in D_2O		Reported value[5]	
position	δH , type (J in Hz)		δH (⊿)
4, 6	2.64-2.82, m, 4H	2.83, m, 4H	
7	3.46, s, 2H	3.58, s, 2H	-0.12
8	3.58, s, 3H	3.70, s, 3H	-0.12
9	4.15, s, 2H	4.09, s, 2H	0.06
12	4.26, d, 1H (3.7Hz)	4.11, d, 1H (5.0 Hz)	0.15
13	4.33, m, 1H (6.3, 3.8 Hz)	4.33, d of q, 1H (5.0, 6.4 Hz)	0
14	1.15, d, 3H (6.4 Hz)	1.25, d, 3H (6.4)	-0.1

Porphyla-334

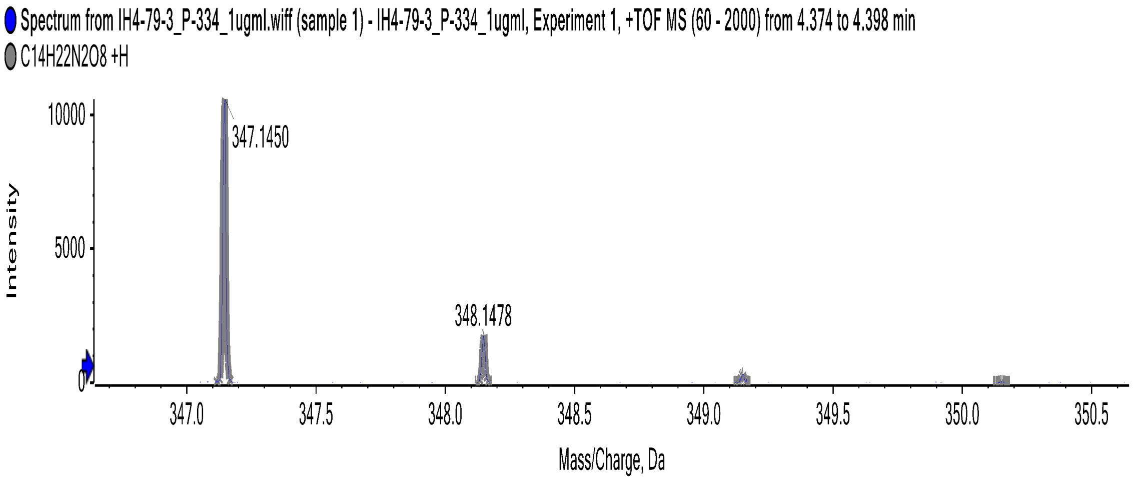

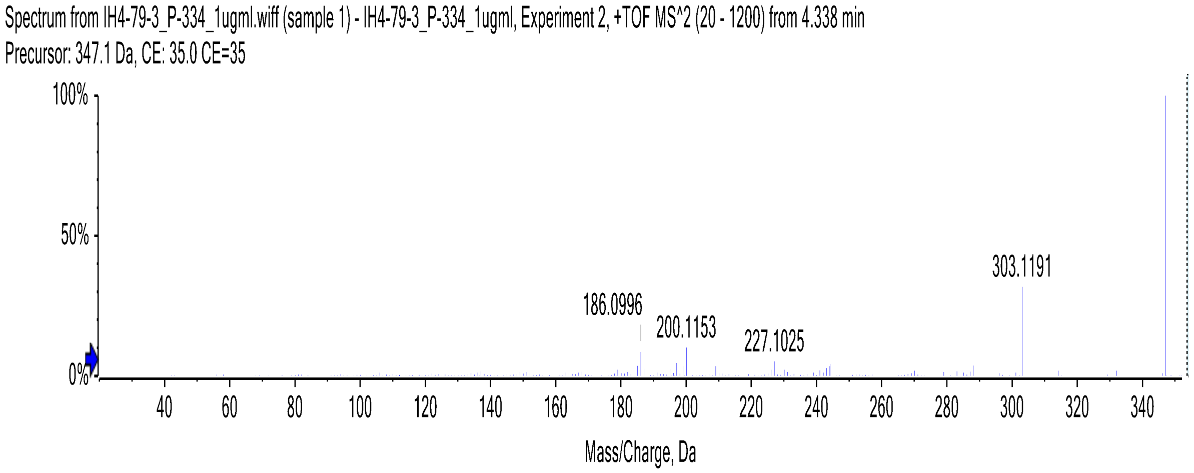

Palythenic acid (Z)

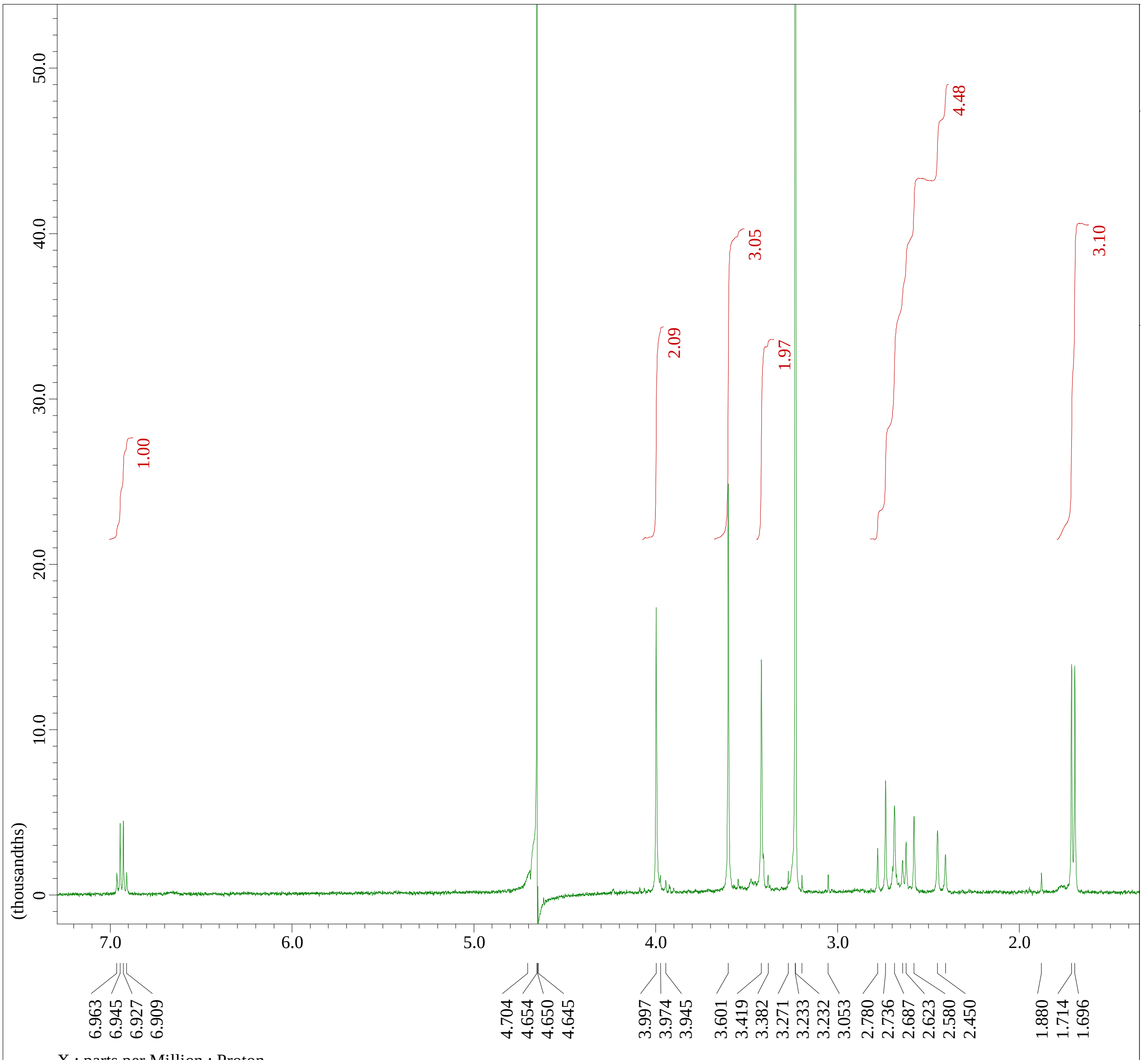

Palythenic acid in D_2O			Reference in D_2O
position	δH, type (J in Hz)	Reported value[6]	δH (⊿)
CH_2-4*	2.43, 2.60, ABq, 2H (17)	2.52, 2.69, ABq, 2H (17)	
CH_2-6*	2.67, 2.76, ABq, 2H (17)	2.72, 2.85, ABq, 2H (17)	
CH_2-7	3.49, s, 2H	3.52, s, 2H	-0.03
CH_3-8	3.60, s, 3H	3.70, s, 3H	-0.1
CH_2-9	4.00, s, 2H	4.05, s, 2H	-0.05
CH-11	6.95, d, 1H (7.3)	6.82, q, 1H (7.2)	0.13
CH_3-14	1.71, d, 3H (7.3)	1.77, d, 1H (7.2)	-0.06

*Assignments may be interchanged.

Palythenic acid

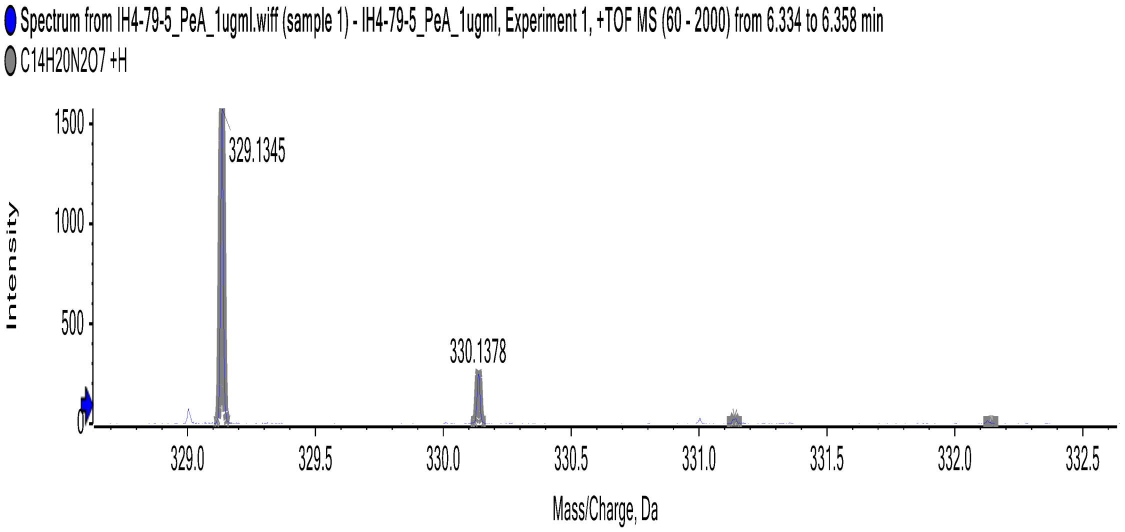

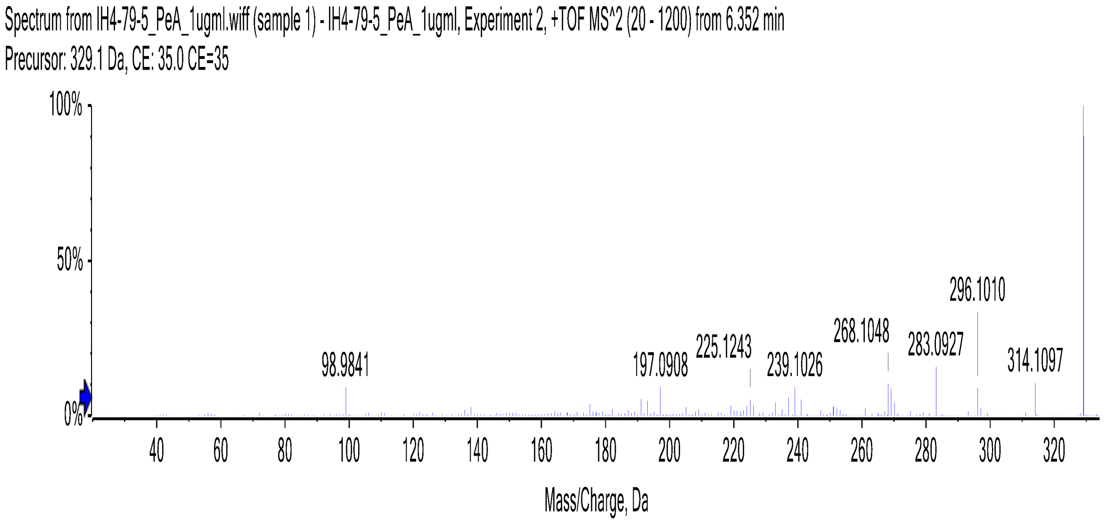

HRESIMS data (upper) with molecular ion (MH^+) calcd for $C_{14}H_{21}N_2O_7$, 329.1349; found 329.1345, and MS/MS data (lower) for palythenic acid.

Usujirene/palythene 7:3 mixture

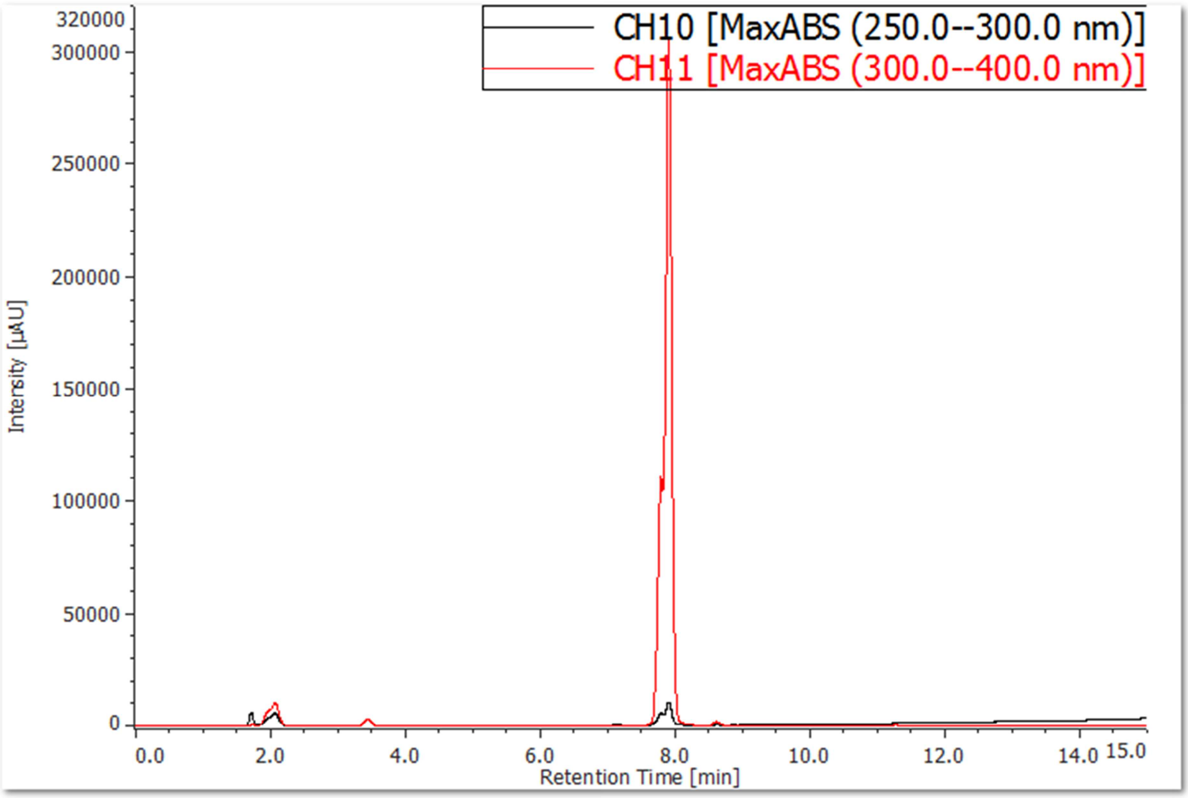

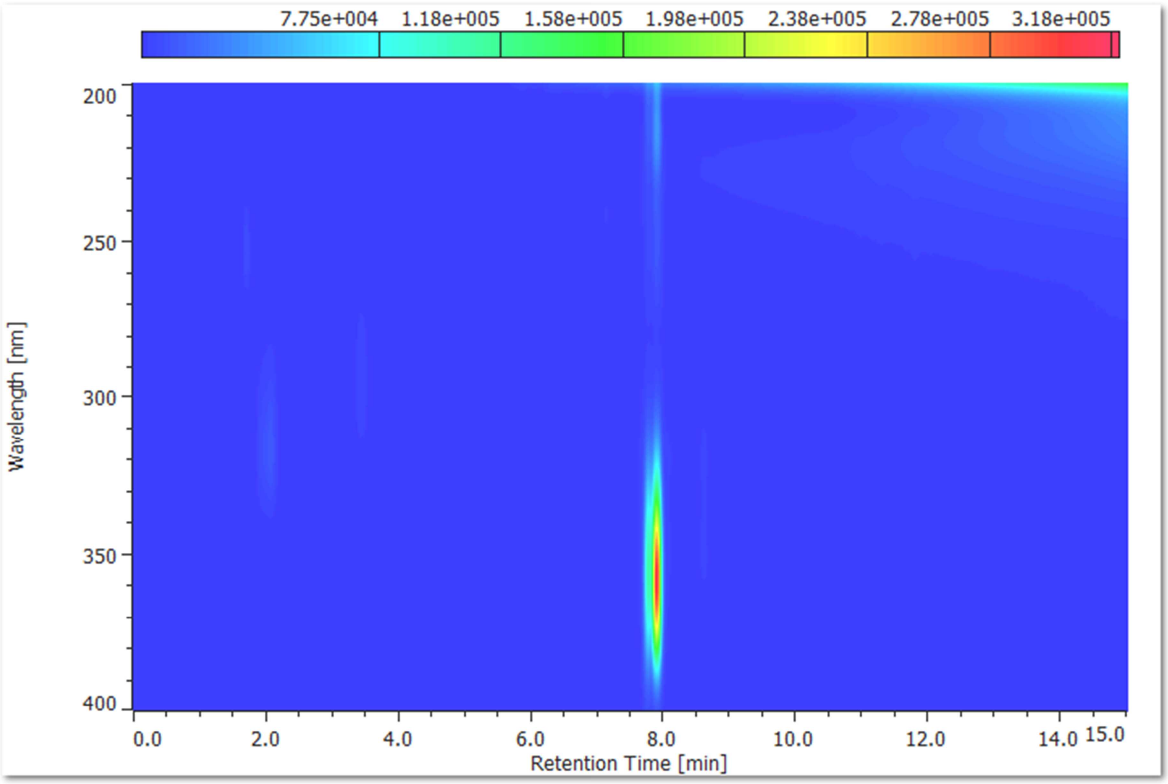

Usujirene/palythene 7:3 mixture

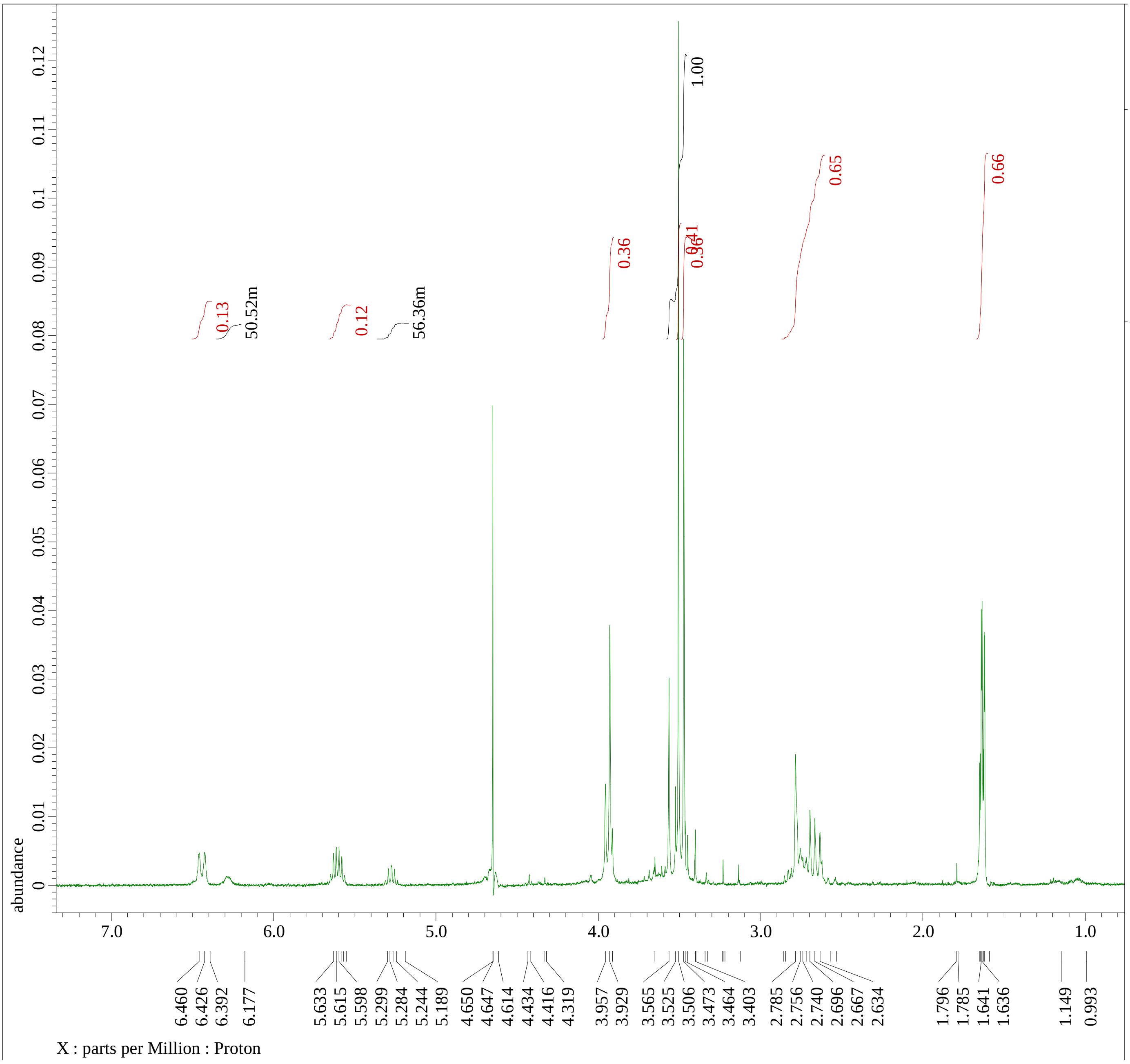

Palythene

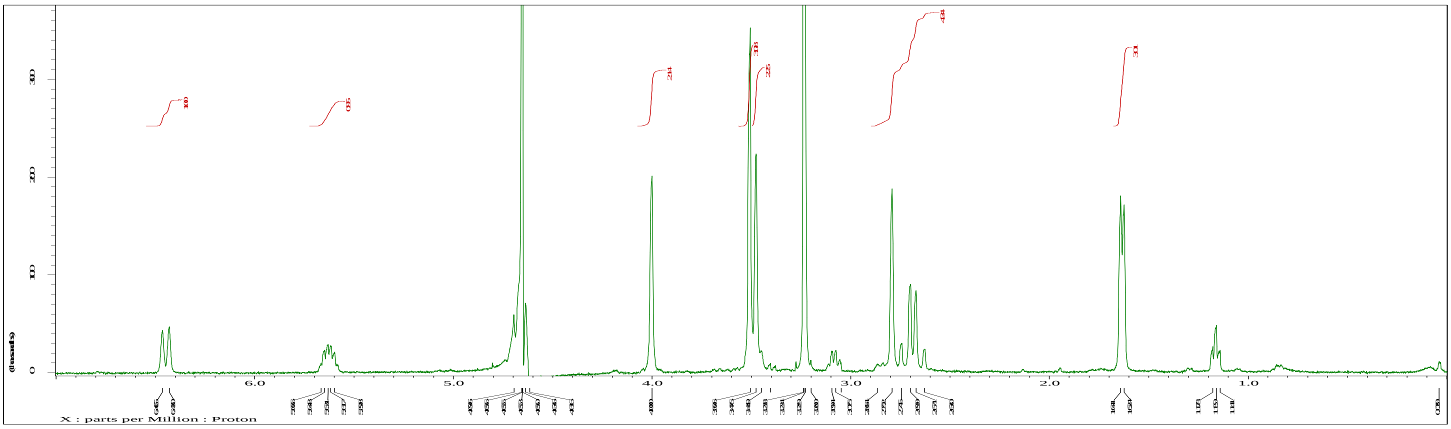

Usujirene

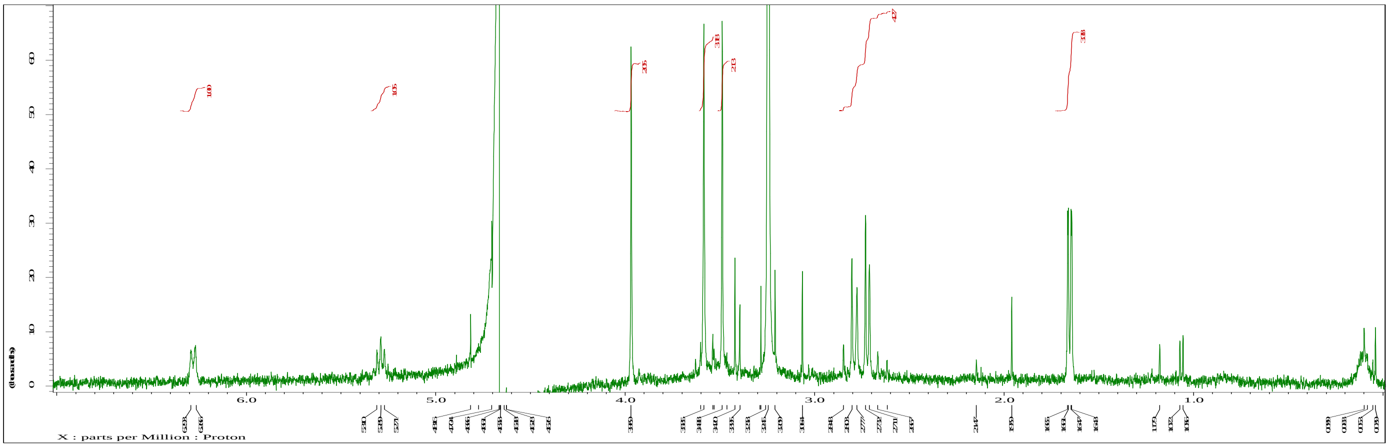

Palythene(usujirene) in D_2O			
position	δ_H , type (J in Hz)	Reported values for palythene[3]	
CH_2-4*	2.66,2.72 ABq (18Hz)(2.63-2.83, 4H, m)	2.96, ABq, 4H (17Hz)	
CH_2-6*	2.79, s		
CH_2-7	3.47 (3.49), s	3.66, s, 2H	-0.19
CH_3-8	3.51 (3.59), s	3.71, s, 3H	-0.2
CH_2-9	3.93(3.97), s	4.11, s, 2H	-0.18
CH-11	6.44(6.28br), d, (13.9Hz)	6.58, brd, 1H	-0.14
CH-12	5.60(5.28), sep (7.2 Hz)	5.75, dq, 1H	-0.15
CH_3-13	1.63 (1.64), dd (6.8, 2 Hz)	1.88, dd, 3H	-0.25
*Assignments may be interchanged.			

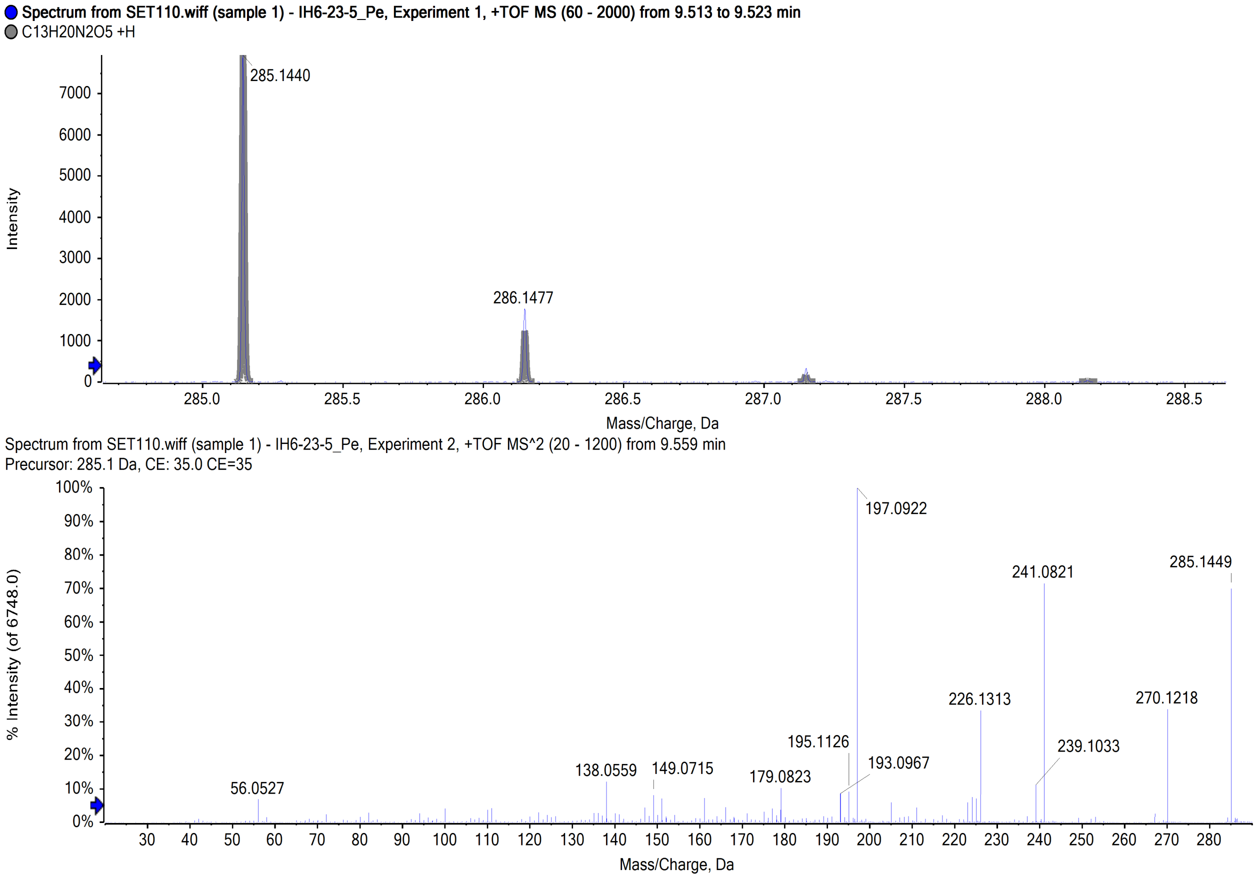

HRESIMS data (upper) with molecular ion (MH^+) calcd for $C_{13}H_{21}N_2O_5$, 285.1451; found 285. 1440, and MS/MS data (lower) for usujirene/palythene.

Mycosporine-2-glycine

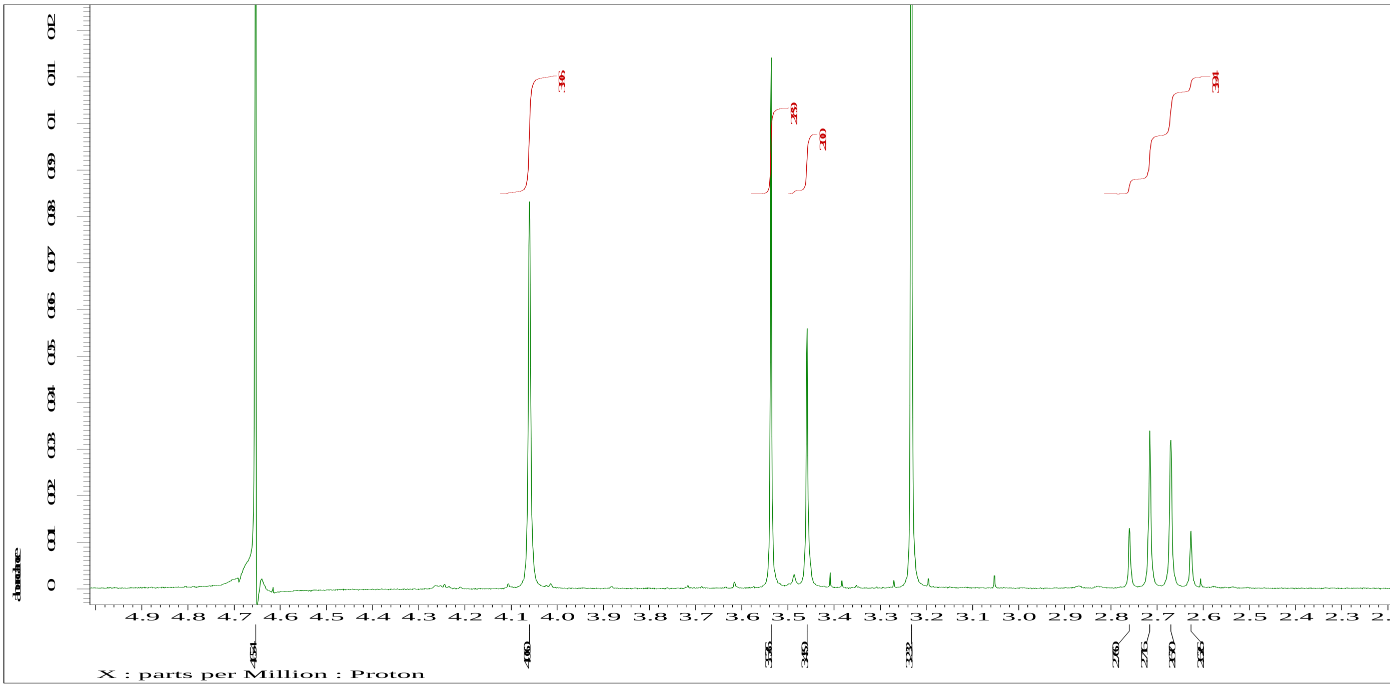

Mycosporine-2-Gly in D_2O		Reported values[7]	
position	δH , type (J in Hz)		δH (⊿)
CH_2-4, 6	2.65, d, 2H (17.7)	2.77, d, 2H (17)	-0.12
	2.74, d, 2H (17.4)	2.86, d, 2H (17)	-0.12
CH_2-7	3.46, s, 2H	3.60, s, 2H	-0.14
CH_3-8	3.53, s, 3H	3.68, s, 3H	-0.15
CH_2-9	4.05, s, 4H	4.06, s, 4H	-0.01

Mycosporine-2-glycine

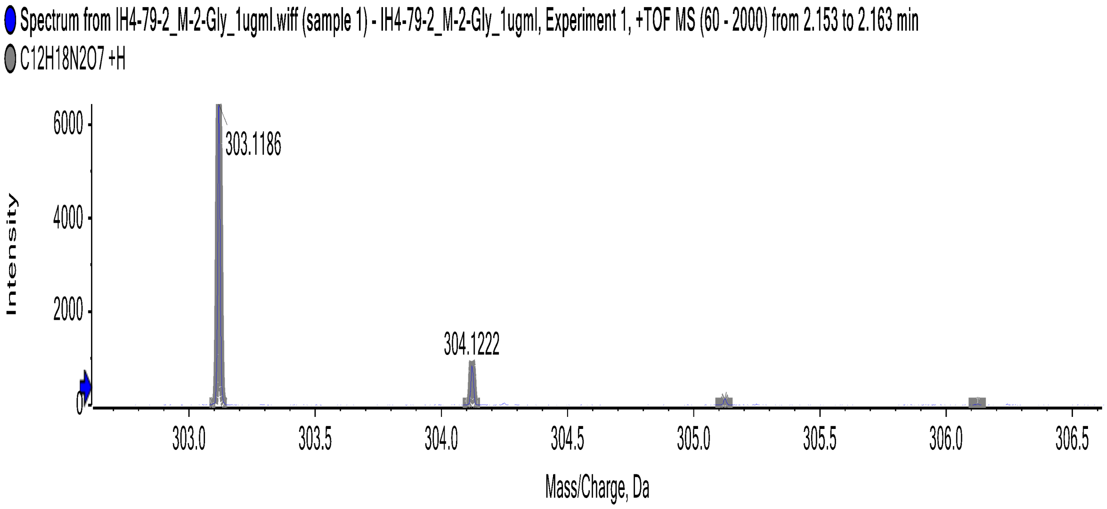

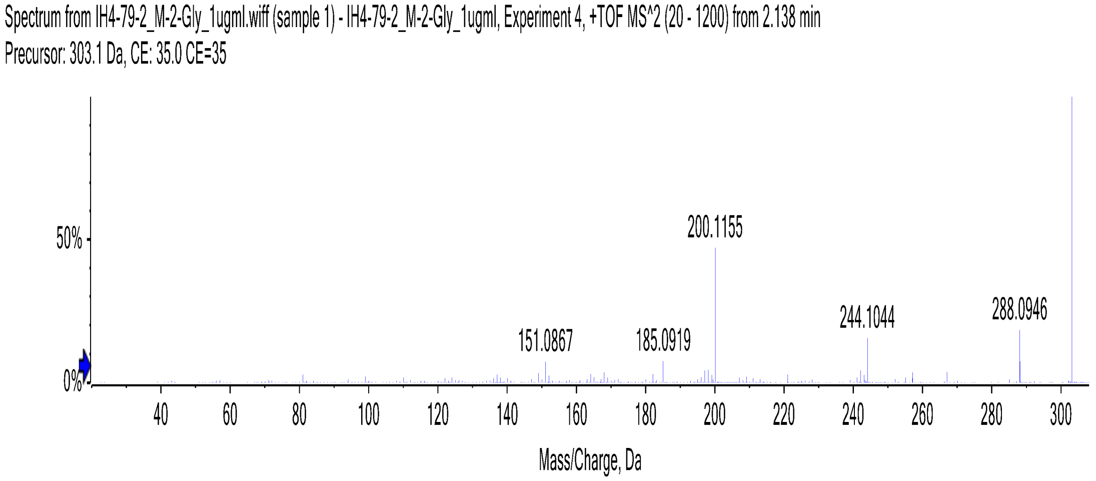

HRESIMS data (upper) with molecular ion (MH^+) calcd for $C_{12}H_{19}N_2O_7$, 303.1187; found 303.1186, and MS/MS data (lower) for mycosiporine-2-glycine.

Palythine

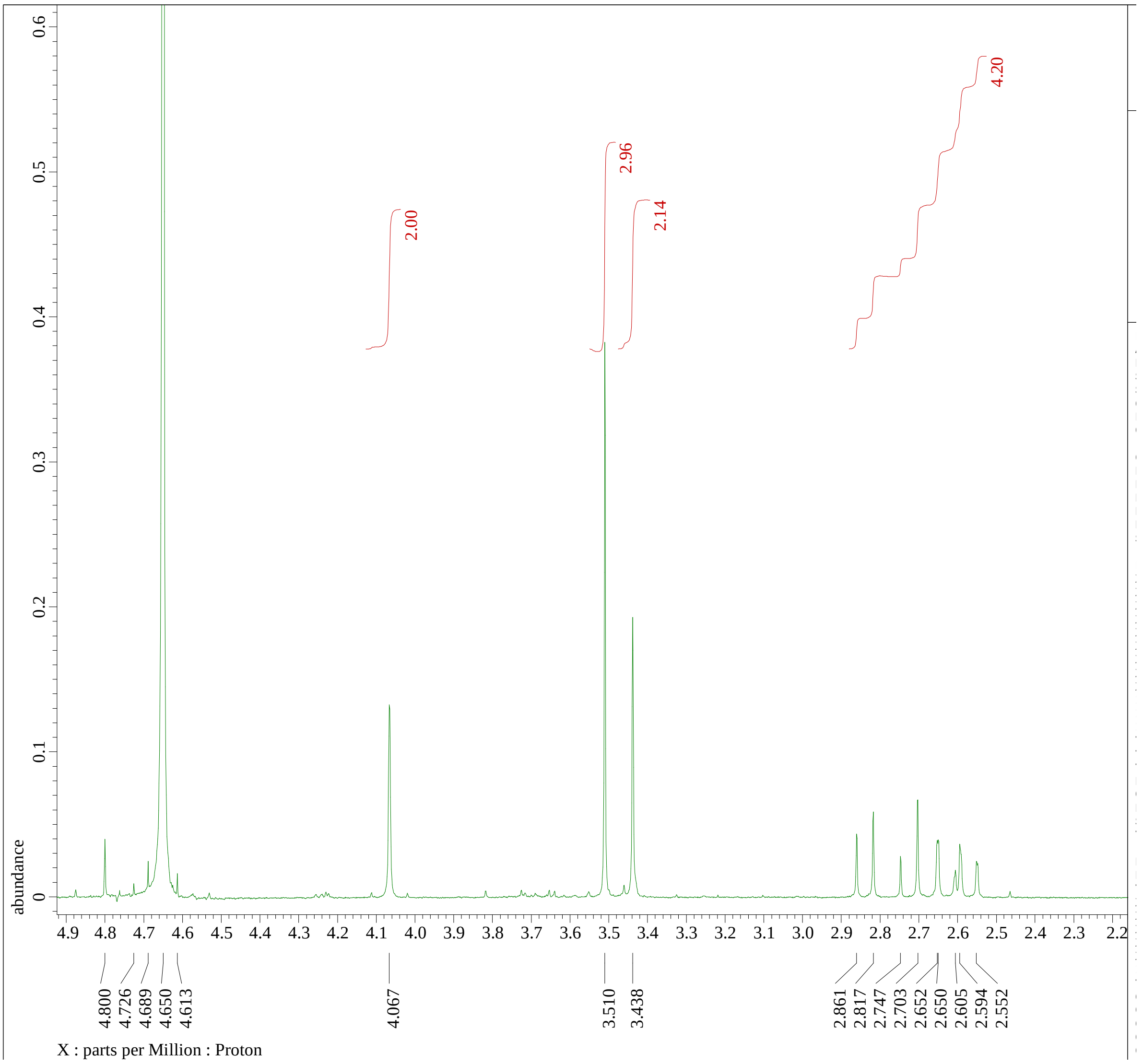

Palythine in D_2O		Reported values[8]	
position	δH , type (J in Hz)	δH , type (J in Hz)	δH (⊿)
CH_2-4*	2.57,2.84 ABq (17)	2.69,3.00 ABq (17)	-0.12,-0.16
CH_2-6*	2.64,2.72 ABq (17)	2.73,2.92 ABq (17)	-0.09,-0.20
CH_2-7	3.44, s, 2H	3.60, s, 2H	-0.17
CH_3-8	3.51, s, 3H	3.67, s, 3H	-0.16
CH_2-9	4.07, s, 2H	4.06, s, 2H	-0.01

*Assignments may be interchanged.

Palythine

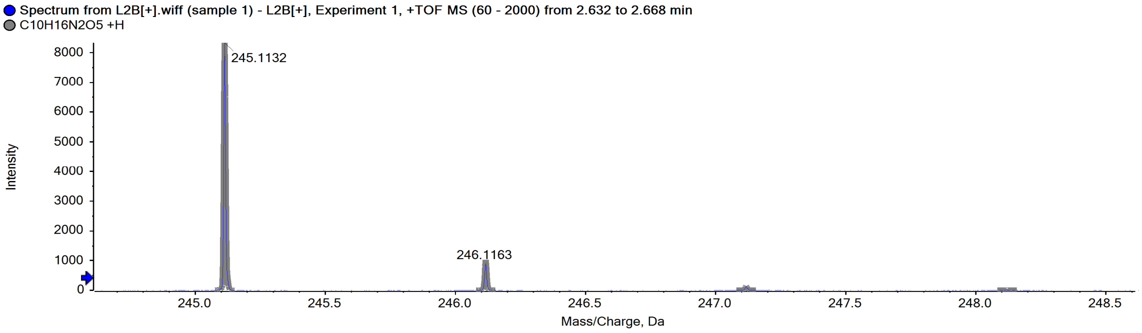

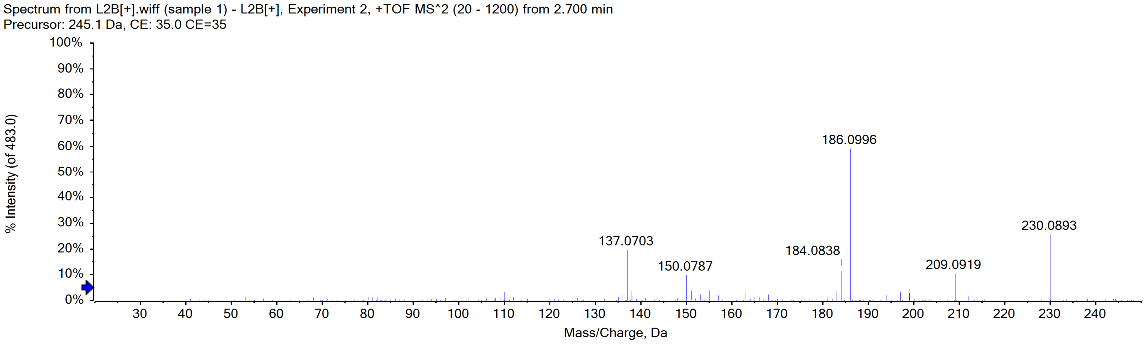

HRESIMS data (upper) with molecular ion (MH^+) Calcd for $C_{10}H_{17}N_2O_5$, 245.1132; found 245.1132, and MS/MS data (lower) for palythine.

References

1. Ito, S., Isolation and structure of a mycosporine from the zoanthidian Palythoa tuberculosa. *Tetrahedron Lett.* **1977,** *18*, 2429-2430.

2. Hartmann, A.; Gostner, J.; Fuchs, J. E.; Chaita, E.; Aligiannis, N.; Skaltsounis, L.; Ganzera, M., Inhibition of collagenase by mycosporine-like amino acids from marine sources. *Planta Med.* **2015,** *81* (10), 813-820.

3. Takano, S.; Uemura, D.; Hirata, Y., Isolation and structure of two new amino acids, palythinol and palythene, from the zoanthid palythoatubercolosa. *Tetrahedron Lett.* **1978,** *19* (49), 4909-4912.

4. Nakamura, H.; Kobayashi, J.-i.; Hirata, Y., Isolation and structure of a 330 nm UV-absorbing substance, asterina-330 from the starfish Asterina pectinifera. *Chem. Lett.* **1981,** *10* (10), 1413-1414.

5. Takano, S.; Nakanishi, A.; Uemura, D.; Hirata, Y., Isolation and structure of a 334 nm UV-absorbing substance, porphyra-334 from the red alga Porphyra tenera Kjellman. *Chem. Lett.* **1979,** *8* (4), 419-420.

6. Kobayashi, J. i.; Nakamura, H.; Hirata, Y., Isolation and structure of a UV-absorbing substances 337 from the ascidian haloctnthia Roretzi. *Tetrahedron Lett.* **1981,** *22* (31), 3001-3002.

7. Kedar, L.; Kashman, Y.; Oren, A., Mycosporine-2-glycine is the major mycosporine-like amino acid in a unicellular cyanobacterium (Euhalothece sp.) isolated from a gypsum crust in a hypersaline saltern pond. *FEMS Microbiol. Lett.* **2002,** *208* (2), 233-237.

8. Takano, S., Isolation and structure of a new amino acid, palythine, from the zoanthid Palythoa tuberculosa. *Tetrahedron Lett.* **1978,** *26*, 2299-300.
